# Tuning the Surface State and Zeta Potential of Aged CuO Nanoparticles by Additive‐Free Surface Treatments as Guided by XPS

**DOI:** 10.1002/adma.202513030

**Published:** 2025-10-26

**Authors:** Anastasia S. Batenkova, Bastian Rheingans, Claudia Cancellieri, Lars P.H. Jeurgens

**Affiliations:** ^1^ Laboratory for Joining Technologies and Corrosion Empa ‐ Swiss Federal Laboratories for Materials Science and Technology Dübendorf CH‐8600 Switzerland; ^2^ Department of Chemistry and Applied Biosciences ETHZ ‐ Federal Institute of Technology Zurich Zürich CH‐8093 Switzerland

**Keywords:** CuO nanoparticles; nanoparticle aging, nanopowders, surface charge, XPS, zeta‐potential

## Abstract

Broad application of nanoparticles, for e.g., catalysis, antimicrobial coatings, medicine, electronics, batteries, and nanojoining technologies, is hindered by their limited shelf‐life due to ambient aging. This study traces the changes in the surface chemistry of nanoparticles during atmospheric aging by monitoring the changes in the C 1*s*, O 1*s*, Cu 2*p* core‐level spectra using XPS. The atmospheric aging of nanoparticles proceeds via hydroxylation and the adsorption of organic‐O surface species, which introduces defects in the subsurface region. The position and shape of the main Cu 2*p*_3/2_ peak and its satellite provide a sensitive fingerprint of the nanoparticle surface state, while the O 1*s* spectra reveal the formation of subsurface O defect species. Air annealing at 300°C and room‐temperature ozonation provide additive‐free, environmentally friendly surface treatments that can convert arbitrarily aged surface back to well‐defined (pristine) CuO nanoparticle surface states. Thus, reproducible dispersion characteristics of CuO nanoparticles in ethanol can be obtained independently of the aging history. These findings help to extend the shelf‐life of nanoparticles and enable reproducible processing routes for CuO nanopowders to be established for advancing nanotechnologies such as nanoporous oxide membranes, catalysts, functional coatings, and batteries.

## Graphical abstract

Aging of CuO nanoparticles (NP) involves a surface reconstruction induced by hydroxylation and concurrent organic‐O adsorption, which introduces O defects in the NP subsurface region. The defective surface results in a diffuse surface space region, as reflected by a low ζ‐potential of the aged NPs in ethanol solvent. Air‐annealing or ozonation of the aged NPs successfully restores the pristine CuO surface state, resulting in a much higher ζ‐potential which enables the fabrication of nanoporous CuO coatings by electrophoretic deposition.

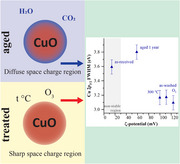

## 1 Introduction

Nanomaterials offer unique physicochemical properties and enhanced reactivity due to the relatively high volume fraction of atoms associated with surfaces and interfaces (e.g. phase and grain boundaries), which have local chemical environments that deviate from the bulk.^[^ ^1^, ^2^, ^3^, ^4^ ^]^ For example, as a rule of thumb, more than 10% of the atoms constitute the surface of a 10nm diameter metallic nanoparticle.^[^ ^5^ ^]^ Many functional properties of nanoparticles are thus dominated by the electronic structure of the NP surface, which opens up great potential for surface engineering of nanoparticles (NPs), as applied in e.g., catalysis,^[^ ^6^, ^7^, ^8^ ^]^ batteries,^[^ ^9^ ^]^ magnetic storage,^[^ ^10^, ^11^, ^12^ ^]^ printable electronics,^[^ ^12^, ^13^, ^14^ ^]^ 3D printing,^[^ ^15^ ^]^ sensing,^[^ ^16^ ^]^ medicine,^[^ ^17^, ^18^, ^19^ ^]^ wastewater treatment,^[^ ^20^ ^]^ optics,^[^ ^21^ ^]^ functional coatings^[^ ^22^, ^23^ ^]^ and nanojoining technologies.^[^ ^12^, ^24^, ^25^, ^26^ ^]^ Unfortunately, the actual surface state and properties of NP nanopowders (as constituted of aggregates and agglomerates of primary nanocrystallites, resulting in very high specific surface areas (SSAs)^[^ ^27^ ^]^) are extremely sensitive to the storage and processing conditions.^[^ ^5^, ^28^ ^]^ Most nanopowders undergo continuous and non‐uniform (i.e., heterogeneous) changes in their surface chemistry during atmospheric aging, depending on, e.g., temperature, humidity, and gas composition. This results in unpredictable and irreproducible NP surface properties during processing (e.g., reactivity, solubility, dispersability, printability, sinterability).

Aging of synthesized NPs, nanowires and nanoplatelets has been reported in numerous studies.^[^ ^28^, ^29^, ^30^, ^31^, ^32^, ^33^ ^]^ For example, atmospheric exposure of ZnO nanowires results in the overgrowth of ZnCO_3_ by spontaneous surface reactions with CO_2_ and humidity.^[^ ^34^ ^]^ A decrease in the magnetic properties and NP growth of ZnO NPs after one year of aging under ambient conditions has also been reported.^[^ ^35^ ^]^ The electrocatalytic activity of freshly synthesized Pt and Pd NPs decreases when aged in air (as well as at a slower rate in solution).^[^ ^36^ ^]^ Similar results have been obtained for as‐prepared and aged Au NPs.^[^ ^29^ ^]^ An increased solubility of aged CuO and ZnO NPs in aqueous environments, as associated with an enhanced toxicological risk, has also been reported.^[^ ^31^, ^33^ ^]^ The present study demonstrates a deterioration of the dispersion characteristics of CuO NPs in ethanol due to a decrease of the surface charge (ζ‐potential) upon atmospheric aging.

Of course, any spontaneous surface reactions of NPs with the ambient can be blocked by protecting the surface with a thin but dense shell of an chemically‐inert surfactant or additive, as is generally done for air‐sensitive metallic nanoparticles of e.g., Al, Fe, Cu and Ni.^[^ ^37^, ^38^, ^39^, ^40^, ^41^ ^]^ As common practice, various (potentially environmentally hazardous) organic compounds are used for stabilization (i.e., to prevent NP aggregation and agglomeration, while providing protection against ambient aging) in an attempt to control and preserve the surface properties of freshly‐synthesized (further referred to as *pristine*) NPs.^[^ ^36^, ^42^ ^]^ However, thin organic shells around the NPs will result in totally different surface properties and processing behaviors (e.g., aggregation/agglomeration, sintering, solubility, dispersibility, reactivity), even for shell thicknesses as thin as a single monolayer. Moreover, considering the high surface‐to‐volume ratio of NPs, the use of organic additives to stabilize and/or modify the NPs may lead to a relatively high amount of unwanted organic residues and/or reacted carbon compounds after processing. Such carbon residues may not only deteriorate the materials performance, but also create reliability issues in real‐life applications (e.g., surface poisoning in NP catalysis,^[^ ^43^, ^44^ ^]^ insulating Li_2_CO_3_ reaction layers in Li solid‐state batteries,^[^ ^45^ ^]^ resistance to NP sintering). Alternatively, well‐defined surface treatments or surface modifications that circumvent the usage of surface additives have been proposed to counteract the inevitable aging of the surface state of pristine NPs, such as annealing,^[^ ^46^ ^]^ doping,^[^ ^47^ ^]^ quenching,^[^ ^48^ ^]^ and smart adaptation of the synthesis route.^[^ ^44^ ^]^ Likewise, in the present study, different mild and additive‐free surface treatment strategies (i.e., washing, annealing and ozonation) are explored to convert the aged surface state of CuO nanopowders back to a well‐defined pristine surface state, targeting at a reproducible dispersion behavior of the CuO NPs in ethanol solvent (as dictated by the so‐called ζ‐potential ^[^ ^27^ ^]^).

The two most common bulk oxide phases encountered in the Cu─O system are cuprous or copper(I) oxide (i.e., Cu_2_O with a Cu (I)‐valence state and a cubic structure) and cupric or copper(II) oxide (i.e., CuO with a Cu (II) valence state and a monoclinic structure). CuO NPs are applied in various applications, such as catalysis, energy conversion, magnetic storage, antimicrobial coatings, printable electronics and nanojoining technologies.^[^ ^49^, ^50^ ^]^ As a first step toward a solution to extend the shelf‐life of commercial CuO nanopowders and control their surface properties for the aforementioned applications, in‐depth knowledge of the change in the NP surface state during atmospheric aging is needed. On the basis of such knowledge, easily‐scalable and environmentally friendly surface treatments may be developed to restore any arbitrarily aged surface state back to its pristine surface state.

Accordingly, in the present study, changes in the chemical surface states of commercial CuO NP powders during aging under atmospheric conditions were investigated in detail by XPS. It is demonstrated that the shapes of the Cu 2*p*_3/2_ main peak and its respective satellite provide robust and very sensitive fingerprints for tracing subtle changes in the surface state of the CuO nanopowder during aging. As will be discussed in Section 2.3, the satellite structure is characteristic for Cu(II) valence states in pristine CuO, whereas the position and shape of the Cu 2*p*_3/2_ main peak is a sensitive probe of the local chemical Cu─O bonding states ^[^ ^51^, ^52^, ^53^, ^54^ ^]^. Next, additive‐free surface treatments of arbitrarily aged CuO nanopowder batches were conducted (i.e., washing, thermal annealing in air and ozonation at room temperature) for converting the aged surface state back to a well‐defined (pristine) surface state. The applicability of our approach to control a specific surface property of CuO NPs, independent of their aging history, is exemplified by monitoring the ζ‐potential (i.e., surface charge) of aged and surface‐treated CuO NPs dispersed in ethanol solvent. The proposed surface treatments all succeed in increasing the ζ‐potential value of aged CuO nanopowders toward reproducible optimized values. The procedure and strategies developed in this work set an important basis for our ongoing research on the production of nanoporous CuO films by electrophoretic deposition from CuO NP dispersions without the common use of surfactants.^[^ ^55^ ^]^ Moreover, the reported findings provide a foundation for extending the shelf‐life of different kinds of commercial oxide nanopowders (being inexpensive and available in sufficiently large quantities) to allow easy upscaling for applications, such as for nanopaste sinter bonding, catalysis, and ink‐jet printing.

## 2 Results and Discussion

### 2.1 Phase Constitution, Morphology, and Specific Surface Area (SSA) of the As‐Received CuO Nanopowders

**Figure 1:**
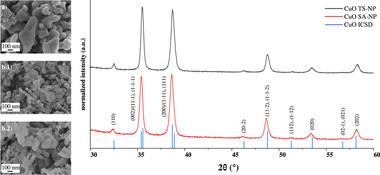
Representative high‐resolution Secondary Electron Microscopy (SEM) micrographs and corresponding XRD diffractograms of a) the as‐received CuO TS‐nanopowders and b) the as‐received CuO SA‐nanopowders (for two different representative batches, denoted as b.1 and b.2). The reflections for bulk CuO (with the respective Miller indices) according to the ICSD database are indicated by the blue lines.

The current study uses two commercial CuO nanopowders purchased from Thermo Scientific and Sigma Aldrich, which are referred to as TS‐NP and SA‐NP, respectively. X‐ray Diffraction (XRD) analysis confirms that the as‐received nanopowders are composed of a single CuO bulk phase (i.e., no other bulk oxide and/or hydroxide bulk phases are detected): see **Figures** **1**a,b. Williamson‐Hall (WH) analysis of the recorded diffractograms (see Table 1 of Section S8, Supporting Information) indicates average crystallite sizes of 60nm for the TS nanopowder and 50nm for the SA nanopowder. These average crystallite sizes are compatible with the NP sizes ranges provided by the suppliers based on TEM analysis (i.e., 20–50nm for TS and ⩽50nm for SA). Selective in‐house TEM analysis of finely dispersed TS and SA NPs (see Figure S1.1, Supporting Information) indicates a broader range of primary NP sizes for the TS nanopowders (as compared to the SA nanopowder). Some primary NPs are constituted of different crystalline domains (“nanograins”), which may result in slight differences between the primary NP size ranges (by TEM) and the primary crystallite size ranges (by XRD). Successive manufacturing and processing steps (and environmental exposure) result in aggregation and/or agglomeration of these primary NPs into larger entities (also also evidenced in Figure S1.1, Supporting Information) with sizes of up to several microns. According to SEM analysis of (non‐dispersed) nanopowders, the as‐received CuO nanopowders are constituted of oval‐ and platelet‐shaped CuO NP assemblies (with slight variations of the morphology between the different batches; see Section 4) with a broad average size range of roughly 40–400nm for TS and 50–200nm for SA: see Figures 1a,b. A platelet morphology of the bulk CuO nanophase indicates that the supplier's synthesis process likely involved a cost‐effective wet chemical synthesis route via a Cu‐hydroxide precursor phase (e.g., obtained by precipitation from a salt solution), which is subsequently transformed by calcination into a CuO phase.^[^ ^27^ ^]^ The BET analysis indicates specific surface areas (SSAs) of 4m^2^g^−1^ and 12.5–14.3m^2^g^−1^ (depending on the batch) for the TS and SA nanopowders, respectively. These in‐house measured values are approximately three times lower than the corresponding SSAs stated by the supplier (i.e., 13.0m^2^g^−1^ for TS and 29.0m^2^g^−1^ for SA). The broader size range and correspondingly lower SSA observed in our in‐house measurements confirm particle agglomeration/aggregation, as well as variations in batch quality of the supplier (resulting in deviations from the specified datasheet).

**Table 1:** Adopted fitting constraints for the chemical shifts and FWHMs of the different Adv‐C 1*s* species with respect to the aliphatic C─C/C─H main peak, as well as of the different O 1*s* species with respect to the O 1*s* main peak for (bulk) CuO: see Figure 4. The corresponding peak components are annotated with capital letters A–J in Figure 4. Note that the C 1*s* spectral contributions from C─C and C─H species, as well as from C─OH and C─O─C species, overlap and cannot be unambiguously separated by peak fitting.^[^ ^60^ ^]^ The same holds for the O 1*s* spectral contributions from defective O (sub)surface ($O_{\mathrm{surf}}^{\delta -}$) and carbonate (CO_3_) species (see Section 2.4).

Chemical Species	Label Figure 4	Peak Position [eV]	Standard deviation	FWHM [eV]
**C 1*s***				
C─C/C─H	A	284.8	–	1.6 + −0.1
C─OH/C─O─C	B	A + 1.4	0.2	A
C═O	C	A + 2.7	0.2	A
O─C═O	D	A + 3.9	0.1	A
CO_3_	E	A + 5.2	0.2	A
**O 1*s***				
$O_{\mathrm{bulk}}$ (CuO lattice)	F	529.6	0.1	1.2 + −0.1
OH	G	F + 0.9	0.1	1.6 + −0.1
$O_{\mathrm{surf}}^{\delta -}$/CO_3_	H	F + 1.9	0.1	G
O_Adv_ (organic‐O)	I	F + 3.1	0.2	G
H_2_O	J	F + 4.2	0.2	G

**Figure 2:**
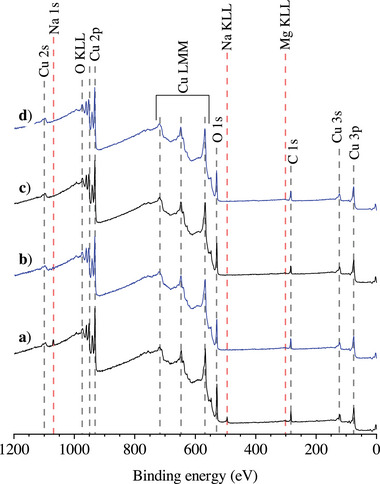
Measured XPS survey spectra of a) the as‐received CuO TS‐NPs, (b) the as‐received CuO SA‐NPs, (c) the as‐washed CuO TS‐NPs and (d) the as‐washed CuO SA‐NPs.

The surface chemistry of the as‐received CuO NPs (in‐house stored under Ar, prior to the washing step; see Section Materials and Analysis) was analyzed by XPS: see **Figure** **2**. The XPS survey spectrum from the TS‐NPs in Figure 2a indicates the presence of Na and a lesser amount of Mg impurities on the CuO TS‐NP surface. In addition, practically unavoidable adventitious carbon surface species are detected on both types of nanopowders, as will be discussed in more detail Section 2.3. The Na impurities on the TS‐NPs were effectively removed by the default washing procedure (see Section 2.5): compare Figure 2a,b with Figure 2c,d. Still, a small contamination of the TS‐NPs by Mg remained after washing. The overall smaller particle size of the CuO SA‐NPs and the absence of initial surface contaminants renders it our preferred starting material for further experiments. Accordingly, all results in the following refer to those obtained from different batches of as‐washed and/or surface‐treated CuO SA‐nanopowder.

### 2.2 General Challenges for the XPS Analysis of Nanopowders

**Figure 3:**
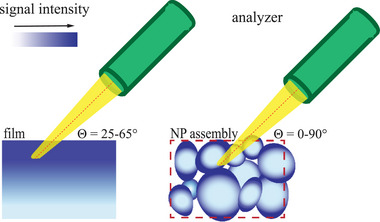
Schematic illustration of the probed XPS analysis volume for a flat surface as compared to a randomly packed NP assembly with open micro‐ and nano‐porosity. The dark blue areas in the illustrations indicate the probed volumes of elastically scattered and non‐scattered photoelectrons emitted from a thin film and a given NP assembly. These probed volumes span core and surface regions with different inelastic scattering cross sections. For a flat surface, photoelectrons are detected at a defined angular range. In our setup, the analyzer acceptance is (45±20)°, which results in a defined range of detection angles and thus a defined range of analysis depths. For NP assemblies, however, curvature and inhomogeneity further broaden the take‐off angle distribution and thereby the analysis depth range. The probed surface‐to‐volume photoelectron signal intensities from the NP assembly will thus depend on the selected position and area for XPS analysis.

Tracing very small changes of the surface state upon aging by XPS is not as straightforward for nanopowders as for flat surfaces and thin films.^[^ ^56^ ^]^ First, from a safety viewpoint, dry nanopowders should be handled in an under‐pressurized glove box with subsequent transfer for XPS analysis in sealed containers (see Section Materials and Analysis), which is cumbersome in practice. Second, XPS analysis of oxide nanopowders may suffer from differential charging issues, causing e.g., artificial (asymmetric) peak broadening and/or “ghost” peaks.^[^ ^57^ ^]^ Fortunately, as discussed in Section S5 (Supporting Information), no indications for differential charging of the CuO NPs during XPS analysis were observed in the present study. Third, individual NPs tend to agglomerate and aggregate to reduce their surface energy, which typically results in a non‐uniform NP packing density. Photoelectrons emitted from core and surface regions of CuO NP assemblies will experience different inelastic scattering cross‐sections to reach the analyzer, depending on size distribution and packing density of the NPs, as well as on varying degrees of surface contamination by adventitious carbon species throughout the porous nanopowder.^[^ ^5^, ^28^ ^]^ Furthermore, in the case of a flat surface, the photoelectrons will be detected at a defined set of take‐off angles (as defined by the analyzer angular opening, i.e., ±20°; see Section 4), whereas this angular distribution will be much broader for the corresponding nanopowder (≈0°–90°, as indicated in **Figure** **3**). Consequently, depending on the actual NP packing density in the probed volume, the signal ratio originating from surface and core regions of the NP assembly may vary, as schematically illustrated in Figure 3. Hence, for a given nanopowder under study, the absolute photoelectron intensity, as well as the probed average surface‐to‐volume ratio of the NP assembly will depend on the selected position and area for the XPS analysis (see Figure 3). Notably, the X‐ray beam spot used in our measurements is ≈200μm, which is ≈2000 times larger than the typical NP size (50–100nm), which will average out the aforementioned effects. From practical tests, we noted that especially the local packing density of the nanopowder has a noticeable effect on the probed surface‐to‐volume signal ratio.

**Figure 4:**
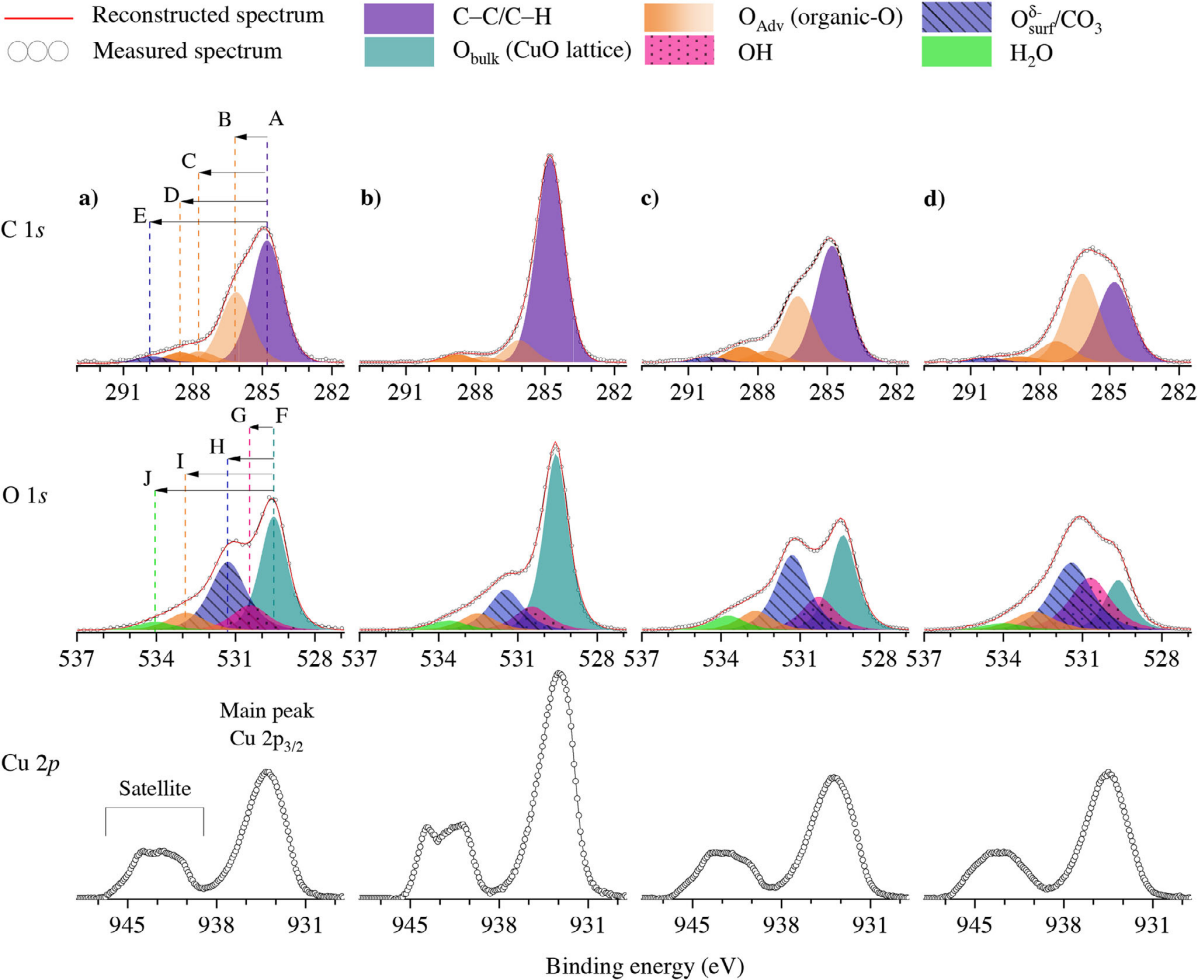
Measured and reconstructed C 1*s* (top row), O 1*s* (second row), and Cu 2*p*_3/2_ (bottom row) XPS spectra of the CuO SA‐NPs for a) the as‐received state, b) the as‐washed state and c,d) after washing and subsequent aging under ambient conditions for (c) 6 months and (d) 2 years. The different chemical species, as resolved by constrained peak fitting of the C 1*s* and O 1*s* regions, are represented by specific colors with the different types of organic‐O species being combined in an orange color scale. The imposed constraints for the chemical shifts and FWHMs of the fitted synthetic main peaks, as labeled from A to J, are reported in Table 1. All spectra shown were normalized by the integrated intensity of the Shirley background corrected spectral envelope to correct for variations in absolute photoelectron intensity, depending on the selected position and area for XPS analysis (see Section 2.2).

To approximately correct for arbitrary variations in absolute photoelectron intensity, depending on the selected position for XPS analysis, all recorded spectra were normalized by the integrated intensity below the Shirley‐background‐corrected peak envelope: see e.g., **Figure** **4**. The relative signal intensities from surface and core regions of the NP assembly will still vary depending on the area and position of the XPS measurement, which hinders a reliable quantification of the (absolute) composition of e.g., surface and bulk species on the basis of the reconstructed Cu 2*p*, C 1*s* and O 1*s* spectral contributions (see Figure 4). Still, C 1*s* spectral contributions may be assumed to originate only from the NP outer surface region (see Section 2.4), which allows a robust *qualitative* evaluation of the cumulative changes in the surface state of the CuO nanopowders upon aging and subsequent surface treatment, as discussed in Sections 2.4 and 2.5.

### 2.3 Peak Fitting and Chemical‐State Assignment of the C 1*s*, O 1*s*, and Cu 2*p* Spectra

A defined washing procedure of the as‐received CuO NPs, as constituted of six alternating washing steps in MilliQ water (with a conductivity of σ = 0.055μSm cm^−1^ at 25°C) and ⩾99.8% absolute ethanol (further denoted **“as‐washed”** for details, see Section Washing, Aging and Surface Treatments) were performed to remove any residual impurities introduced during nanopowder synthesis (e.g., Na, Mg; see Section 2.1). The measured C 1*s*, O 1*s* and Cu 2*p*_3/2_ spectra of the as‐received and as‐washed CuO SA‐NPs, as well as of the as‐washed CuO SA‐NPs after atmospheric aging for 6 months and 2 years (further denoted as **“aged”**), are shown in Figure 4a–d. The different chemical species, as resolved by constrained peak fitting, are each represented by a specific color (with the different types of organic‐O species combined in an orange color scale). It follows that the as‐received, as‐washed and aged CuO nanopowders all have various adventitious carbon (Adv‐C) species on their surfaces, which may partially originate from the synthesis and washing processes, but mainly from the adsorption of gas‐phase species from the ambient.^[^ ^58^ ^]^ In the present study, the measured C 1*s* spectra before and after aging can be well described by accounting for three different classes of Adv‐C surface species, i.e., aliphatic alkyl‐type C─C/C─H, aliphatic organic‐oxygen‐type (e.g., C─OH, C─O─C, C═O, O─C═O) and to a lesser extent carbonate‐type (CO_3_), in accordance with the literature.^[^ ^59^ ^]^ The corresponding chemical shifts and FWHMs of the fitted Adv‐C 1*s* species with respect to the C─C/C─H main peak (at the lowest BE) are tabulated in **Table** **1**.

Fitting the O 1*s* spectrum is known to be challenging due to the relatively large intrinsic O 1*s* line width with respect to the chemical shift(s), which may result in a superposition of different chemical peak components. The O 1*s* component at the lower BE of the O 1*s* spectral envelop (BE ≈529.6 ± 0.1 eV) can be unambiguously assigned to O anions in the core (i.e., bulk lattice) of the CuO NPs,^[^ ^53^ ^]^ further designated as $O_{\mathrm{bulk}}$. An in‐house prepared malachite reference (see Section Materials and Analysis) was used to identify the chemical nature of some of the O 1*s* spectral contributions toward higher BEs. Corresponding synthetic components associated with hydroxide (i.e., OH at 530.4 ± 0.1 eV^[^ ^61^ ^]^) and carbonate species (i.e., CO_3_ at 531.5 ± 0.1 eV^[^ ^62^ ^]^) were resolved from the malachite reference (while introducing the thus‐determined chemical shifts as fitting constraints): see Figure S3.2 (Supporting Information). Considering the very small spectral contribution from carbonate species in the reconstructed C 1*s* spectra (at 290.0 ± 0.1 eV; see Figure 4), the much larger O 1*s* component (at 531.4 ± 0.1 eV) cannot be solely assigned to such carbonate species (even though the O 1*s* photoionization cross‐section is roughly a factor three larger than for C 1*s*). This suggests an overlap of at least two chemical species for the fitted O 1*s* component at 531.5 ± 0.1 eV. Indeed, the assignment of the resolved O 1*s* component at 531.5 ± 0.1 eV is controversially debated in the literature. Some studies attribute this O 1*s* component to chemisorbed oxygen or hydroxide species,^[^ ^53^, ^63^, ^64^ ^]^ while more recent studies assign it to subsurface oxygen at lattice defects or Cu vacancy sites.^[^ ^65^, ^66^ ^]^ As demonstrated in this study (see Section 2.4), the resolved O 1*s* component at 531.5 ± 0.1 eV in the present study originates from a different local chemical state of O atoms in the CuO NP *sub*surface region,^[^ ^70^ ^]^ as invoked by an adsorbate‐induced surface reconstruction:^[^ ^3^, ^66^, ^67^, ^68^, ^69^, ^71^ ^]^ see Section 2.4 for more details. Several XPS studies of metal‐oxide thin films have associated a similar substoichiometric O 1*s* component to a (defective) region of the reconstructed oxide surface.^[^ ^72^, ^73^, ^74^, ^75^ ^]^ Note that such defective O (sub)surface species can only be probed by surface‐sensitive techniques, like XPS, (i.e., they are not detectable by XRD) and will be much more distinct for oxide NPs than for planar, single‐crystalline oxide surfaces, because:^[^ ^3^, ^68^, ^69^, ^71^, ^72^, ^76^, ^77^ ^]^ *i*) NPs have a relatively high volume fraction of surface atoms with unsaturated coordination and deviating local chemical environments, *ii*) have less well‐defined surface facets with many intrinsic surface defects (e.g., vacancies, steps, kinks and ledges) and *iii*) often exhibit lattice distortions as a response to surface stress. Accordingly, we also attribute the resolved O 1*s* component at 531.5 ± 0.1 eV to defective regions (i.e., local compositional and/or structural heterogeneities) of the CuO NP surface region with respect to the bulk‐like NP core region (hereafter referred to as $O_{\mathrm{surf}}^{\delta -}$), as induced by adsorbate‐induced reconstructions upon aging. Finally, two minor O 1*s* components are resolved at 532.7 ± 0.1 and 533.8 ± 0.1 eV, as assigned to O‐containing Adv‐C species (designated as O_Adv_)^[^ ^60^ ^]^ and adsorbed water,^[^ ^64^ ^]^ respectively. The adopted fitting constraints for the chemical shifts and FWHMs of the different O 1*s* species with respect to the O 1*s* main peak for (bulk) CuO,^[^ ^63^ ^]^ are tabulated in Table 1.

**Figure 5:**
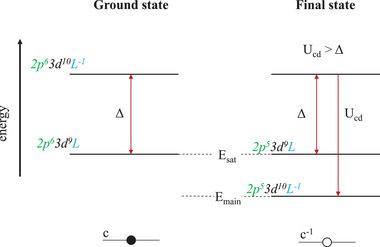
Sketch of the valence configurations used to interpret 2*p* core‐level photoemission in CuO, assuming no coupling between the Cu and O ions. The core hole (*c* ^−1^) on the Cu(II) ion lowers the energy of the 3*d* ^10^ *L* ^−1^ configuration with respect to the 3*d* ^9^ *L* configuration by an amount $U_{\mathrm{cd}}$. Depending on the relative values of $U_{\mathrm{cd}}$ and Δ, either the 3*d* ^10^ *L* ^−1^ configuration is the lowest final state (U_cd_ > Δ) or the 3*d* ^9^ configuration is the lowest (Δ > $U_{\mathrm{cd}}$). Here the case U_cd_ > Δ is shown (which applies to divalent copper compounds). As reproduced from ref. [81].

The measured Cu 2*p*_3/2_ spectra of the as‐received, as‐washed and aged CuO NPs are shown in the bottom row of Figure 4. Spectral reconstruction of the Cu 2*p*_3/2_ − 2*p*_1/2_ doublet is inherently complex due to the superimposition of the 2*p*_3/2_ and 2*p*_1/2_ lines from metallic Cu(0) and oxidic Cu(I) species and the variable fine‐structure of the shake‐up satellite associated with Cu(II) species.^[^ ^53^, ^54^, ^78^, ^79^ ^]^ The presence of such a shake‐up satellite implies that more than one final state can be reached in the photoemission process, as detailed below. The prominent double satellite structure ≈ 9eV BE above the Cu 2*p*_3/2_ main peak is characteristic for CuO (i.e., it is not observed for Cu(0) and Cu(I) species) and arises due to the incomplete *d*‐shell for the Cu(II) valence state.^[^ ^51^, ^52^, ^54^ ^]^ The Cu(II) cation in CuO has a filled ground state configuration of 2*p* ^6^ *d* ^9^ *L*, which mixes with the empty excited state configuration of 2*p* ^6^ *d* ^10^ *L* ^−1^ (where *L* and *L* ^−1^ represent the highest filled oxygen ligand shell before and after transferring a valence electron to the Cu(II) *d*‐shell). The energy difference between the preferred 2*p* ^6^ *d* ^10^ *L* ^−1^ ground state and the 2*p* ^6^ *d* ^9^ *L* excited state is referred to as the charge transfer energy, Δ, as illustrated in **Figure** **5**. The creation of a 2*p* core hole in the Cu(II) cation is accompanied by a strong Coulomb attraction of the *d* ^10^ *L* ^−1^ level (with respect to its initial state) by an amount U_cd_, resulting in two final state configurations, as shown in Figure 5. The first final‐state configuration, 2*p* ^5^3*d* ^9^ *L*, leaves the 3*d* ^9^ ground‐state roughly intact, whereas the second final‐state configuration, 2*p* ^5^3*d* ^10^ *L* ^−1^, involves an electron transfer from the O ligand to the core‐ionized Cu(II) cation (commonly referred to as non‐localized screening). For CuO, $U_{\mathrm{cd}}>\Delta$, and hence the 2*p* ^6^3*d* ^10^ *L* ^−1^ level with the lower energy corresponds to the Cu 2*p*_3/2_ main peak, while the satellite refers to the 2*p* ^5^ *d* ^9^ *L* excited state (as shifted to higher BEs by an amount of U_cd_ − Δ.^[^ ^80^ ^]^)

As follows from the above discussion, the BE position of the Cu 2*p*_3/2_ main peak depends on the ligand interaction (since it involves an electron transfer from the O 2*p* to the Cu 3*d* valence shell), and thus on the distribution of chemical Cu─O bonding states in the NP (sub)surface region, as probed by XPS. Accordingly, the Cu 2*p*_3/2_ region of the Cu 2*p*_3/2_ − 2*p*_1/2_ doublet was analyzed by extracting well‐defined spectral features, in particular, the relative BE positions and FWHMs of the Cu 2*p*_3/2_ main peak and its satellite from the background‐corrected spectra, as exemplified in Section S6 (Supporting Information). The thus‐derived values for the BE and FWHMs of the Cu 2*p*_3/2_ main peak and its satellite are reported for the different CuO NP surface states and reference samples in **Table** **2**. Commonly, the Cu 2*p*_3/2_ region (i.e., main peak plus satellite) is fitted with multiple synthetic peak components for spectral reconstruction.^[^ ^78^, ^79^ ^]^ However, the method adopted in this work turned out to be easier and robust, provided that the presence of metallic Cu(0) species can be excluded.

**Table 2:** Relative BE positions and FWHMs of the Cu 2*p*_3/2_ main peak and its satellite, as extracted from the Cu 2*p*_3/2_ region, according to the procedure outlined in Section S6 (Supporting Information). The reported valence‐state fraction, $\frac{\left[Cu(I)\right]}{\left[Cu(II)\right]}$, was derived using the procedure outlined in ref. [78], while adopting the CuO NP after annealing in synthetic air at 300°C as all‐Cu(II)‐valence‐state reference. The fraction of oxygen (sub)surface species, $f_{O_{\mathrm{surf}}^{\delta -}}$, refers to the area ratio between the resolved surface ($O_{\mathrm{surf}}^{\delta -}$) and bulk ($O_{\mathrm{bulk}}$) O 1*s* peak components, as shown in Figure 4. The tabulated values pertain to the measured Cu 2*p*_3/2_ spectra for the as‐received, as‐washed, aged and surface‐treated CuO SA‐nanopowders, as well as for the in‐house prepared reference samples.

CuO NP Surface State	Cu 2*p*_3/2_ [eV]		Satellite [eV]		$\frac{\left[Cu(I)\right]}{\left[Cu(II)\right]}$	$f_{O_{\mathrm{surf}}^{\delta -}}$	ζ‐potential
	BE	FWHM	BE	FWHM			[mV]
As‐received	934.1	3.6	943.3	4.6	0.00	0.80	14 + −3
As‐washed	933.4	3.2	942.6	4.5	0.07	0.32	111 + −23
Aged 6 month in air	933.8	3.8	942.8	4.9	0.08	0.98	–
Aged 1 year in air	934.0	3.8	943.2	4.8	0.01	1.12	59 + −12
Aged 2 years in air	934.4	3.7	943.6	4.6	0.00	2.05	–
Annealed @300 °C	933.5	3.2	942.6	4.5	0.00	0.30	100 + −21
Ozonated	933.5	3.1	942.5	4.5	0.08	0.40	124 + −26
**Reference Samples** (see Supporting Information)							
CuO thin film	933.6	3.1	942.7	4.6			
Cu(OH)_2_	934.4	4.0	944.2	4.6			
Cu_2_(OH)_2_CO_3_ (malachite)	934.6	3.9	943.2	5.6			

Metallic Cu (0) species can be easily and unambiguously identified from a distinct peak maxima at 921.3 eV (kinetic energy, KE) in the Cu *L*_3_ *M*_4, 5_ *M*_4, 5_ Auger line.^[^ ^53^, ^78^, ^79^, ^82^ ^]^ As follows from Figure S7.1 (Supporting Information), the measured Cu *L*_3_ *M*_4, 5_ *M*_4, 5_ spectra of the studied CuO nanopowders do not show this characteristic peak. Hence, a complete reduction of Cu (II) → Cu (I) → Cu (0)^[^ ^83^ ^]^ by washing or any post‐treatment of the CuO NP can be ruled out in this study. A substantial reduction of Cu (II) → Cu (I) upon aging or treatment can also be excluded, since this would lead to a pronounced shift of the main peak toward lower kinetic energy and the appearance of a characteristic shoulder at ≈922 eV (as observed for Cu (I) compounds like Cu_2_O; see Figure S7.1 (Supporting Information) and Refs. [78, 79, 82]). However, a slight reduction of Cu (II) → Cu (I) valence states is observed in specific cases, as discussed in Section 2.5. All CuO nanopowders studied exhibit peak maxima toward higher kinetic energies and have a characteristic peak shape, as for Cu (II) in CuO.^[^ ^78^, ^79^, ^82^ ^]^ Interestingly, we observe a distinct shift of the Cu *L*_3_ *M*_4, 5_ *M*_4, 5_ peak maximum after two‐years aging toward that of the malachite reference (see also Section 2.4): see Figure S7.1 (Supporting Information).

### 2.4 Surface States of the CuO NPs after Washing and Subsequent Aging

**Figure 6:**
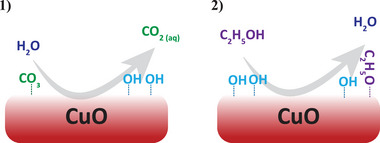
Sketch of the washing treatment consisting of alternating washing steps in MilliQ water and ethanol: see Section 2.5). 1) Protons, present in water, participate in an ion‐exchange interaction between surface impurities on the CuO surface and the bulk solvent, which, e.g., facilitates the removal of carbonate species, as well as Na and Mg impurities (not shown here). 2) Rinsing of the hydroxylated surface with ethanol results in the substitution of OH^−^ groups with ethoxy‐species, which are desorbed upon drying.

As reflected in Figure 4, the Adv‐C surface species on the CuO NPs are composed of C─C/C─H and C─OH/C─O─C bonding states, independent of their history. Comparison of the reconstructed C 1*s* spectra in Figures 4a and 4b indicates that the washing treatment also significantly reduces the surface fractions of organic‐O (i.e., C─OH/C─O─C) species with respect to C─C/C─H species). Strikingly, the washing treatment even suppresses the O 1*s* spectral contribution attributed to $O_{\mathrm{surf}}^{\delta -}$ species with respect to $O_{\mathrm{bulk}}$ species; see corresponding areal fractions, $f_{O_{\mathrm{surf}}^{\delta -}}$, in Table 2. This suggests that the washing procedure largely restores the “pristine” composition and structure of the CuO NP (sub)surface region by the removal of foreign impurities, organic‐O and associated $O_{\mathrm{surf}}^{\delta -}$ defect species. The underlying mechanisms associated with alternating washing steps in MilliQ water and ethanol on the ionic exchanges at the CuO NP surface are schematically illustrated in **Figure** **6**. Water readily participates in an ion‐exchange interaction between surface impurities on the CuO surface and the bulk solvent (e.g., facilitating the dissolution of Na and Mg impurities, as well as carbonate species), while ethanol dehydroxylates the surface, leaving adsorbed ethoxy ions.^[^ ^84^, ^85^ ^]^ Subsequent drying, as well as evacuation down to UHV for XPS analysis, led to the desorption of volatile organic species, resulting in a much cleaner and (largely) dehydroxylated surface.

Subsequent aging of freshly‐washed CuO NPs reactivates surface hydroxylation with renewed formation of organic‐O and associated $O_{\mathrm{surf}}^{\delta -}$ species: compare Figure 4c,d. As discussed above, surface hydroxyl species readily react with Adv‐C surface species, forming organic‐O adsorbate species. After two years of aging, the CuO NP surface region is dominated by hydroxyl, organic‐O, and associated $O_{\mathrm{surf}}^{\delta -}$ surface species (with a minor contribution from CO_3_ species). The minor O 1*s* component at 533.8 ± 0.1 eV due to chemisorbed water^[^ ^64^ ^]^ is roughly independent of the history of the CuO nanopowder. Notably, a humidity of 40% during ambient exposure is sufficient to form a thin layer of water on the surface of CuO NPs^[^ ^87^ ^]^ and its reaction with CO_2_ may lead to the formation of azurite or malachite phases, as observed in this study after prolonged aging. A qualitative comparison of the reconstructed C 1*s* and O 1*s* spectra after washing and subsequent aging indicates a strong correlation between the relative spectral contributions from hydroxyl, organic‐O, and $O_{\mathrm{surf}}^{\delta -}$ species.

Surface reconstructions of ionic compounds, such as oxides, are very common and thermodynamically driven to lower the surface energy by compensating for surface polarity.^[^ ^67^, ^72^ ^]^ Such surface reconstructions critically depend on the crystallographic orientation and chemical termination of the surface plane (e.g., the degree of hydroxylation, segregation and the presence of adsorbed surface species),^[^ ^3^, ^66^, ^67^, ^68^, ^69^, ^71^, ^72^ ^]^ being much more pronounced for oxide NPs as compared to thin oxide films (see Section 2.3). For example, anhydrous oxide surfaces are known to reconstruct to lower their surface energy by hydrolysis of metal‐oxygen surface bonds (i.e., by surface hydroxylation).^[^ ^72^, ^88^ ^]^ The C 1*s* and O 1*s* spectral reconstructions indicate that aging proceeds by surface hydroxylation and accompanied formation of organic‐O adsorbate species, which induce a defective local chemical state of O in the NP (sub)surface region. The adsorbate‐induced changes of the CuO NP surface upon aging are also evidenced from the evolutions of the positions, widths and shapes of the Cu 2*p*_3/2_ main peak and its Cu(II)‐satellite: see Figure 4 and Table 2. The measured Cu 2*p*_3/2_ spectra all show the satellite peak characteristic for Cu(II) valence states at the higher BE side of the Cu 2*p*_3/2_ main peak, which complies with a CuO bulk phase as indicated by XRD (see Figure 2). However, the characteristic shape of the satellite peak for pristine CuO, having two distinct two‐peak maxima,^[^ ^78^ ^]^ is only observed after washing; the characteristic two‐peak‐maxima feature disappears upon aging, developing a featureless satellite shape indicative of a broader distribution of Cu─O chemical bonding states (see Section 2.3). The as‐received CuO nanopowders also have a rather featureless satellite peak shape, which indicates that the supplied nanopowders have already experienced some degree of aging. As described in the earlier works by Okada & Kotani ^[^ ^89^ ^]^, the two‐peak‐maxima in the satellite structure arise from the asymmetry in hybridization between the Cu 3*d* and O 2*p* valence orbitals for CuO, having four O ligands in the *ab* plane and two O ligands along the *c*‐axis (with the local symmetry of the *D*_4*h*_ point group). The disappearance of the two‐peak‐maxima upon aging in Figure 4 thus suggests a change of the defined [CuO_6_] nearest‐neighbour coordination sphere in CuO by an adsorbate‐induced surface reconstruction.

**Figure 7:**
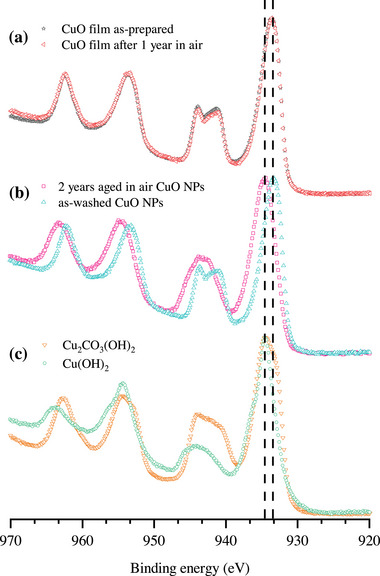
Comparison of selected sets of as‐measured XPS spectra of the Cu 2*p*_3/2_ main peak and its satellite, as recorded from: a) the freshly‐prepared CuO reference film prior to air exposure and after one year of aging in air; b) the CuO NPs directly after washing and after aging for two years in air; c) the commercial Cu(OH)_2_ and the freshly‐prepared Cu_2_(OH)_2_CO_3_ (malachite) reference powders. Note that all spectra were normalized to the peak maximum to ease visual comparison.

Selected sets of as‐measured Cu 2*p* spectra are shown in **Figures** **7**a–c, which visualize the aforementioned changes in the shape of the satellite peak upon aging for the CuO NPs with respect to the measured Cu 2*p* reference spectra of the CuO thin film, Cu(OH)_2_ and Cu_2_(OH)_2_CO_3_ (malachite). As discussed in Section 2.3, the position and FWHM of the Cu 2*p*_3/2_ main peak are more sensitive fingerprints for subtle changes in the Cu─O chemical bonding states than the accompanied shape changes of the satellite peak. Indeed, a modification of Cu─O chemical bonding states in the NP surface region upon aging is evidenced by a distinct broadening and accompanied peak shift of the main peak toward higher BEs: see Figure 7b and Table 2. For the as‐washed state, the Cu 2*p*_3/2_ main peak is positioned at a BE of 933.3 ± 0.1 eV. Upon aging, the main line shifts toward the BE position for the Cu(OH)_2_ reference (at 934.6 ± 0.1 eV, in accord with ref. [78]). However, a prominent shoulder at the lower BE side of the Cu 2*p*_3/2_ main peak, as evident for the Cu(OH)_2_ reference (see Figure 7), does not evolve after prolonged aging. This can be rationalized by the fact that newly formed hydroxyl groups readily react with Adv‐C surface species forming organic‐O adsorbate species (see Section 2.3). The respective FWHM of the Cu 2*p*_3/2_ main peak varies by ≈0.7eV for the different surface states (see Table 2). The corresponding energy separation between the main line and the satellite (i.e., U_cd_ − Δ; see Section 2.3) varies within the experimental error for all studied surface states and thus provides a less robust fingerprint for aging (although a general trend of an increasing energy separation with aging time can be seen). The observed increases of the BE position and FWHM of the Cu 2*p*_3/2_ main peak upon aging comply with a gradual surface hydroxylation with concurrent formation of organic‐O adsorbate species, which both affect the Cu─O bonding states and associated charge distributions in the CuO subsurface region (giving rise to the appearance of $O_{\mathrm{surf}}^{\delta -}$ species). Strikingly, a distinct chemical shift of the Cu 2*p*_3/2_ main peak of the CuO nanopowders toward the respective peak position of malachite is observed between one‐year and two‐years of aging (as for the Cu *L*_3_ *M*_4, 5_ *M*_4, 5_ peak in Figure S7.1, Supporting Information). These findings indicate an induction period (i.e., a kinetic barrier) for the thermodynamically driven, sluggish phase transformation sequence^[^ ^88^ ^]^ of the pristine CuO NPs toward malachite.

Strikingly, the CuO thin‐film reference does not seem to experience any changes in the shapes and positions of the main line and its satellite upon aging in air for one year: compare Figure 7a,b. This clearly demonstrates the enhanced catalytic reactivity of NP surfaces as compared to thin‐ films due to the much high volume fraction of surface atoms with local chemical environments that deviate from the bulk^[^ ^1^, ^2^, ^3^, ^4^ ^]^ (see Section 2.3). Accordingly, the surface properties of CuO NPs are much more sensitive to ambient aging as compared to CuO thin films (and also with respect to micro‐sized CuO powders). The spectral differences between the as‐washed and two‐year‐aged CuO NPs in Figure 7b are obvious. The spectra of the Cu(OH)_2_ and Cu_2_(OH)_2_CO_3_ (malachite) reference samples are compared in Figure 7c and mainly show a difference in their splitting energy U_cd_ − Δ (see Section 2.3).

As discussed in Section 2.3, the Cu 2*p*_3/2_ satellite structure originates solely from Cu(II) valence states, whereas the respective main peak may contain a mixture of spectral contributions from Cu(0), Cu(I) and/or Cu(II) valence states. Hence, the area ratio between the Cu 2*p*_3/2_ main peak and its satellite provides a sensitive measure for tracing changes in the Cu valence state distribution upon aging. The presence of metallic Cu(0) species for the as‐received, aged and treated CuO NPs can be ruled out in the present study (see Section 2.3). Accordingly, the valence‐state fraction, $\frac{\left[Cu(I)\right]}{\left[Cu(II)\right]}$, was calculated from the measured Cu 2*p*_3/2_ spectra, following the procedure outlined in ref. [78], while assuming that the CuO NPs after annealing at 300°C in synthetic air (see Section 2.5) only contain Cu(II) valence states (i.e., $\frac{\left[Cu(I)\right]}{\left[Cu(II)\right]}\equiv 0$). Thermal oxidation at 300°C is known to stabilize CuO over Cu_2_O,^[^ ^83^ ^]^ which motivates our choice to adopt the CuO NP after annealing at 300°C in synthetic air as all‐Cu(II) reference.^[^ ^86^ ^]^ As follows from the calculated values for $\frac{\left[Cu(I)\right]}{\left[Cu(II)\right]}$ in Table 2, the as‐washed (and ozonated; see Section 2.5) CuO NPs contain some Cu (I) valence states. This implies that during the ethanol washing step, a small fraction of Cu (II) ions is partially reduced to Cu(I). Adsorbed ethoxides can undergo oxidative dehydrogenation to form acetaldehyde and H_2_O, creating an oxygen vacancy and partially reducing the metal.^[^ ^85^, ^90^ ^]^ In this regard, it is noted that in the absence of ice‐cooling during the washing steps (see Section Washing, Aging and Surface Treatments), the temperature in the ultrasonic bath rises to 45°C or even higher, which resulted in even higher Cu(I) valence state fractions $\left(\frac{\left[Cu(I)\right]}{\left[Cu(II)\right]}=0.20\right)$. Hence, the Cu(II)‐to‐Cu(I) reduction during the ethanol washing steps is a thermally activated process, which can be suppressed by cooling, thus obtaining a reproducible “as‐washed” surface state. After six months of aging of freshly washed nanopowder, a few Cu(I) valence states are still detected, but they get fully annihilated after longer aging times.

### 2.5 Surface States of the Aged CuO NPs After Room‐Temperature Ozonation and Air‐Annealing

**Figure 8:**
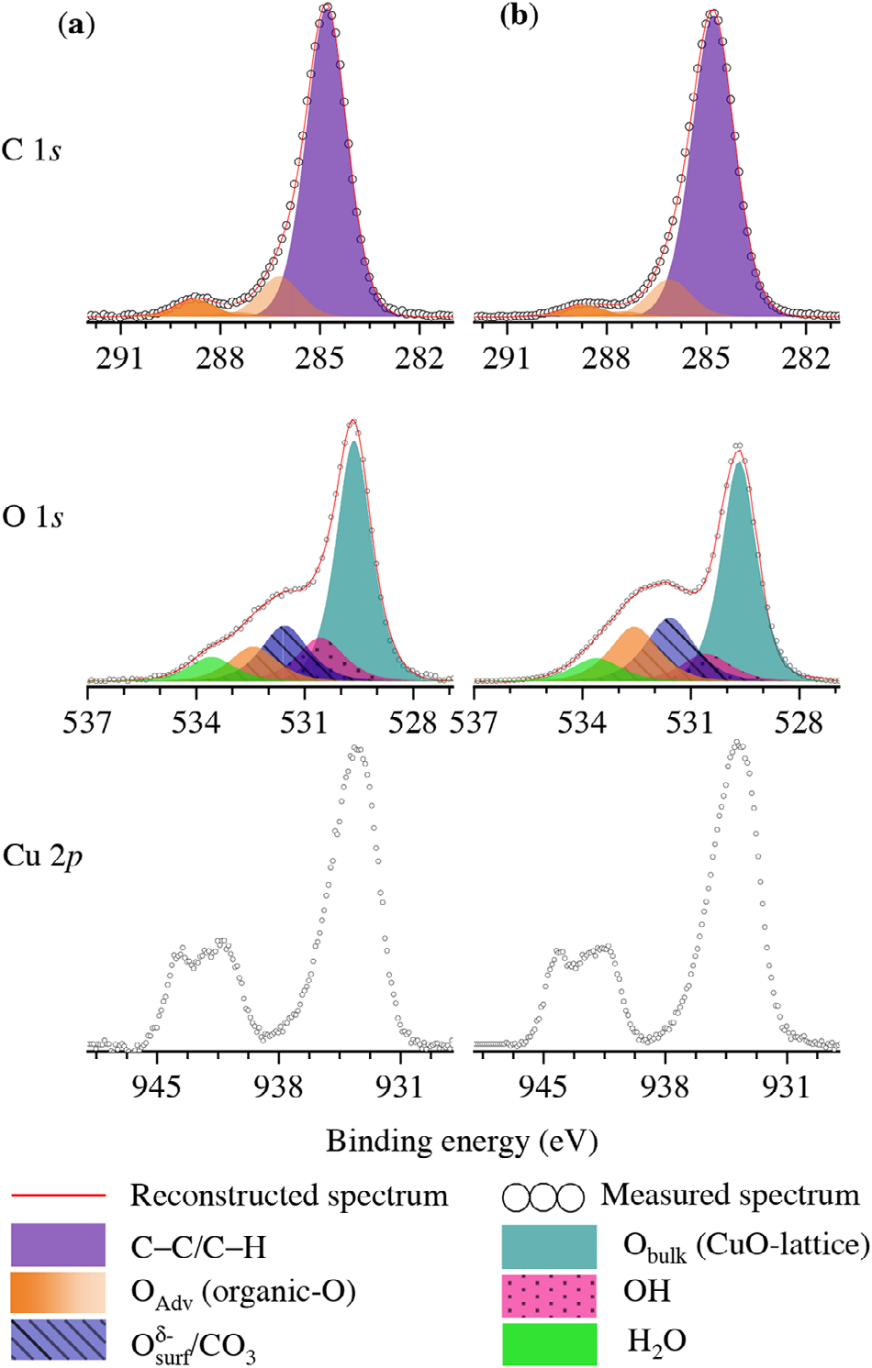
Measured and reconstructed C 1*s* (top row), O 1*s* (second row), and Cu 2*p*_3/2_ (bottom row) XPS spectra of the as‐received (i.e., arbitrarily aged) CuO SA‐NPs after a default washing treatment followed by a) an annealing treatment for 10 min in synthetic air at 300°C, or b) exposure to an ozonated air atmosphere for 30 min at room temperature. The different chemical species, as resolved by constrained peak fitting of the C 1*s* and O 1*s* regions, are represented by the same legend colors as in Figure 4. The imposed constraints for the chemical shifts and FWHMs of the fitted synthetic main peaks (as labeled from A to J in Figure 4) are reported in Table 1. All spectra shown were normalized by the integrated intensity of the Shirley background corrected spectral envelope to correct for variations in absolute photoelectron intensity, depending on the selected position and area for XPS analysis (see Figure 2.2).

Annealing in an inert, oxidizing or reducing atmosphere, as well as room‐temperature exposure to reactive gaseous species like ozone, are common methods to invoke phase transformations and/or selectively modify the surface state of a given material.^[^ ^91^ ^]^ Accordingly, two additive‐free, cost‐effective surface treatments of as‐received (i.e., arbitrarily‐aged; see Section 2.4) CuO nanopowder batches were conducted in an attempt to reproducibly convert any aged surface state back to a well‐defined “pristine” CuO surface state. The two optimized surface treatments both constitute a default washing treatment followed by (see Section Washing, Aging and Surface Treatments): *i*) an annealing treatment for 10 min in synthetic air at 300°C (denoted as **“annealed”**), or *ii*) exposure to an ozonated air atmosphere for 30 min at room temperature (denoted as **“ozonated”**). The measured and reconstructed C 1*s*, O 1*s* and Cu 2*p*_3/2_ spectra of the aged CuO SA‐NPs after the aforementioned surface treatments are shown in **Figure** **8**a,b, respectively. It follows that both surface treatments result in surface states that are very similar to that of the as‐washed CuO NP: compare Figures 4b and 8a,b. This raises the question whether such surface treatments are actually necessary for restoring the pristine CuO surface state or if our default washing treatment suffices to restore the pristine surface state for an arbitrarily aged CuO batch, as will be discussed in more detail below.

Comparison of the surface states of the as‐washed and the surface‐treated CuO NPs indicates that the conducted surface treatments do not introduce new types of adventitious C 1 s surface species (compare Figures 4b and 8). In this regard, it is emphasized that O_3_ should mainly react with unsaturated hydrocarbons surface species,^[^ ^92^ ^]^ which are not abundant in the present study. For the ozonation treatment, oxygen surface species generated on the surface due to O_3_ decomposition induce a slight increase of $O_{\mathrm{surf}}^{\delta -}$ species in O 1s (see Table 2 and Figure 8b).^[^ ^93^ ^]^ While the ozonation treatment is performed at room temperature, the air‐annealing treatment is conducted at 300°C. Accordingly, the ozonation treatment will mainly act on the outer surface of the CuO NP, whereas the oxidation treatment 300°C can also thermally dehydrate the surface and thermally activate atomic rearrangements within the subsurface region of the CuO NP (i.e., by activating solid‐state diffusion). The air‐annealing treatment indeed seems more effective in reducing the overall amount of Adv‐C surface species (according to a qualitative comparison of the measured XPS survey spectra before and after treatment): see Figure 8.

**Figure 9:**
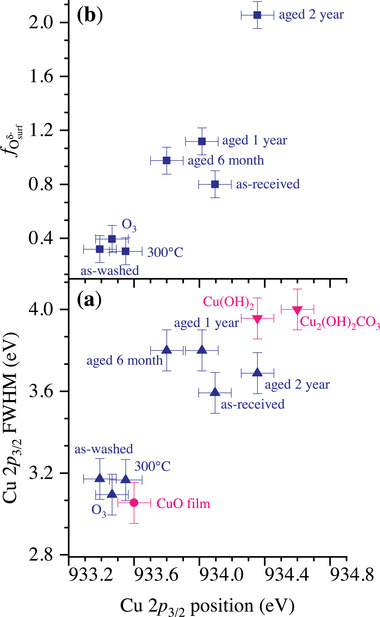
BE of the Cu 2*p*_3/2_ main peak as a function of a) the FWHM of the respective Cu 2p_3/2_ main peak, and b) the respective fraction of oxygen (sub)surface species, $f_{O_{\mathrm{surf}}^{\delta -}}$, as resolved from the reconstructed O 1*s* spectra (see Figures 4 and 8). The corresponding values for the in‐house prepared reference samples (see Table 2) are also plotted in (a) (*pink markers*), indicating a sluggish phase transformation sequence^[^ ^88^ ^]^ of the pristine CuO (sub)surface toward copper hydroxide and malachite upon aging.

The very similar surface states for the as‐washed and surface‐treated CuO NPs are also evidenced by plotting the BE position of the Cu 2*p*_3/2_ main peak as function of either the corresponding FWHM or $O_{\mathrm{surf}}^{\delta -}$ fraction: see **Figure** **9**a,b, respectively. Both plots evidence a clear division between two different types of data sets, forming one data cloud for the as‐washed and surface‐treated CuO NPs (at low BEs and low FWHMs, respectively, at low BEs and low $O_{\mathrm{surf}}^{\delta -}$) and another data cloud for the aged CuO NPs (at high BEs and high FWHMs, respectively, at high BEs and high $O_{\mathrm{surf}}^{\delta -}$). The two‐year‐aged surface state seems to produce an outlier in Figure 9b, which can be rationalized by the formation of carbonate surface species after prolonged aging which further shifts the Cu 2*p*_3/2_ main peak to higher BEs, while also increasing the concentration of unsaturated O subsurface species (see Section 2.4).

**Figure 10:**
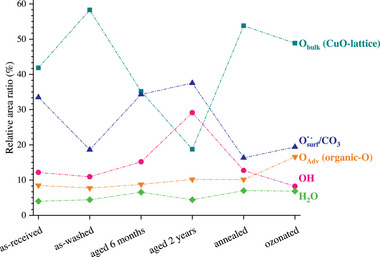
Relative spectral contributions from the different O species (i.e., $O_{\mathrm{bulk}}$, $O_{\mathrm{surf}}^{\delta -}$ / CO_3_, OH, O_Adv_ and H_2_O), as resolved from the reconstructed O 1*s* spectra (see Figures 4 and 8) for the as‐received, as‐washed, aged and surface‐treated CuO NPs. The relative area ratio corresponds to the integrated area of the resolved peak component with respect to the total area of the O 1*s* spectral envelope. Evidently, the as‐washed and surface‐treated (i.e., ozonated and air‐annealed) CuO NPs have by far the largest spectral contribution from $O_{\mathrm{bulk}}$ species. Increasing contributions from OH and $O_{\mathrm{surf}}^{\delta -}$ / CO_3_ (and to a lesser extent from O_Adv_) upon aging can be noted. The adsorbed water content is about independent of the surface state. Slight differences between the annealed and ozonated surface states can be resolved (see Section 2.5).

The bulk references for CuO, copper hydroxide (Cu(OH)_2_) and malachite (Cu_2_(OH)_2_CO_3_) are also shown in Figure 9a. It follows that the CuO thin film corresponds to the data for the freshly washed and surface‐treated samples, while the Cu(OH)_2_ and Cu_2_(OH)_2_CO_3_ reference phases represent extremes of the data set for the aged surface states (i.e., having the highest Cu 2*p*_3/2_ BE and FWHM values). This trend complies with our previous statement (see Section 2.4) that aging of the CuO NPs proceeds by a sluggish phase transformation sequence of the pristine CuO (sub)surface toward copper hydroxide and malachite. Still, the spread of the individual data points within a given data set exceeds the estimated error bars, as is most distinct for the aged surface states. To reveal underlying (subtle) differences in surface state between the as‐washed, ozonated and air‐annealed CuO NPs, the relative spectral contributions from the different O species, as resolved from the reconstructed O 1*s* spectra (see Figures 4 and 8) are plotted in **Figure** **10**.

As follows from Figure 10, the as‐washed and surface‐treated (i.e., ozonated and air‐annealed) CuO NPs by far have the largest spectral contribution from $O_{\mathrm{bulk}}$ species (with correspondingly small spectral contributions from OH, O_Adv_, and $O_{\mathrm{surf}}^{\delta -}$ species). Strikingly, the ozonation treatment noticeably increases the spectral contributions from O_Adv_ and $O_{\mathrm{surf}}^{\delta -}$ species as compared to the air‐annealing treatment. This suggests that O_3_ species readily react with different active sites on the CuO surface, generating reactive oxygen species (ROS) capable of forming organic‐O species.^[^ ^94^ ^]^ It can thus be concluded that the thermal treatment in synthetic air is more effective in removing Adv‐C surface contamination, as well as in restoring the defective oxide surface region by (thermally activated) atomic rearrangements. The adsorbed water content is about the same for all surface states indicating that the CuO nanopowders all readsorb some water upon (humid) air exposure after surface dehydrydation by the ethanol washing and/or thermal annealing treatment.

The pronounced Cu 2*p*_3/2_ satellite peak for the ozonated and annealed surface states in Figure 8 indicates a dominance of Cu(II) valence states for all surface‐treated CuO NPs, in accord with a CuO bulk phase constitution by XRD (see Figure 1). The corresponding valence‐state fractions, $\frac{\left[Cu(I)\right]}{\left[Cu(II)\right]}$, as calculated from the Cu 2*p*_3/2_ region, are reported in Table 2; their values equal 0.07 for the as‐washed and 0.08 for the ozonated CuO NPs. These Cu(I) valence state fractions are significantly higher as compared to the CuO NPs after annealing at 300°C in synthetic air (selected as all‐Cu(II) reference, i.e., $\frac{\left[Cu(I)\right]}{\left[Cu(II)\right]}\equiv 0.00$; see Section 2.4). This implies that a main difference between the as‐washed, ozonated and annealed CuO NP arises from a different Cu(I) valence state fraction in the (sub)surface region. As discussed in Section 2.4, the washing procedure not only removes Adv‐C surface species, but may also partially reduce Cu(II) surface‐adjacent cations to a Cu(I) valence state. A subsequent ozonation treatment at room temperature mainly acts on the outer surface (see above) and thus does not oxidizes these Cu(I) subsurface species back to the Cu(II) valence state. On the contrary, the ozonation treatment even seems to slightly increase the fraction of Cu(I) species in the probed CuO NP (sub)surface region, which hints at the enhanced formation of organic‐O species (as discussed above). In summary, only the surface treatment in an oxidizing atmosphere at elevated temperatures (*here*: 300°C) produces a clean and all‐Cu(II) surface state of the CuO NPs. Thermally activated diffusion within the subsurface region of the CuO NP is a prerequisite to transfer defective Cu(I) (sub)surface valence states back to Cu(II) valence states (and also to more effectively remove Adv‐C surface species).

It may be speculated that thermal annealing at 300°C could induce aggregation (i.e., “fusing”) of CuO NPs, thereby reducing the SSA of the air‐annealed CuO NP. Accordingly, an optimum annealing temperature of 300°C with a duration time of 10 min was carefully evaluated and selected to hinder NP aggregation and grain growth, while still effectively reducing hydroxyl, organic‐O and associated $O_{\mathrm{surf}}^{\delta -}$ surface species. For example, BET analyses indicated that synthetic‐air‐annealing at 300°C for 10min resulted in only a slight increase in NP size with a negligible reduction in SSA (from 14.3 to 13.3m^2^g^−1^), while still successfully restoring the pristine CuO surface. As follows from ref. [83], an oxidation temperature of 300°C is high enough to stabilize the CuO bulk phase, but not induce any other phase transformations (see also Section S8, Supporting Information).

### 2.6 Relation Between CuO NP Surface State and its ζ‐Potential in Ethanol Solvent

ζ‐potential measurements provide an indirect estimate of the surface charge of NPs in solution and are commonly used to assess the dispersion stability of NP solutions, for example, of CuO NPs in ethanol solvent.^[^ ^27^, ^55^ ^]^ The ζ‐potential of a charged particle is usually obtained from its electrophoretic mobility, µ,^[^ ^95^, ^96^ ^]^ and accordingly defined at the “shear‐plane” where the liquid velocity relative to the particle velocity becomes zero.^[^ ^95^ ^]^ For aqueous systems, a high (absolute) ζ‐potential of, say, larger than | ± 30| mV, indicates strong electrostatic repulsion between the NPs in solution, stabilizing a colloidal system. Lower (absolute) ζ‐potential values indicate weaker repulsive interactions, which can lead to particle aggregation and sedimentation. In the framework of our research, we envisage cost‐effective, scalable, green synthesis of micro‐meter‐thick, nanoporous CuO coatings and membranes by electrophoretic deposition (EPD) from stable CuO NP dispersions in ethanol solvent.^[^ ^55^ ^]^ Notably, the EPD process cannot be performed using aqueous‐based CuO dispersions, because the applied external voltages by far exceed the equilibrium potential for water splitting. In this study, we experimentally defined the ζ‐potential range of CuO NPs in ethanol that is suitable for EPD.

**Figure 11:**
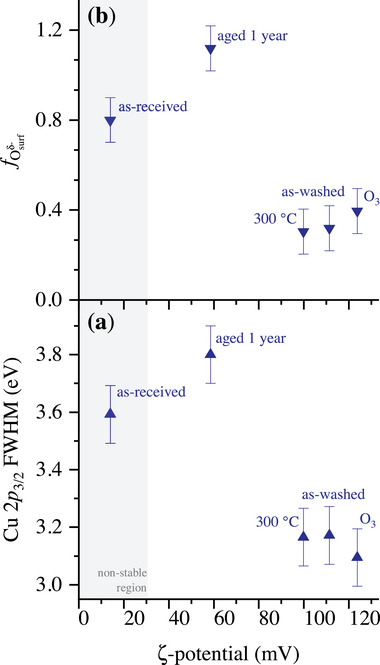
Measured ζ‐potential of the as‐received, as‐washed, aged and surface‐treated CuO NPs in ethanol solution plotted as a function of a) the FWHM of the Cu 2*p*_3/2_ main peak and b) the fraction of (sub)surface $O_{\mathrm{surf}}^{\delta -}$ species, $f_{O_{\mathrm{surf}}^{\delta -}}$, as resolved from the reconstructed O 1*s* spectra (see Figures 4 and 8).

Any dipoles or ionic groups on the CuO NP surface, as induced by surface species (e.g., surfactants, hydroxyl groups and/or Adv‐C species) and/or space‐charge regions originating from defective (sub)surface species (e.g., Cu(I) valence states and/or $O_{\mathrm{surf}}^{\delta -}$ species), will affect the formation of the interfacial double layers and thereby the measured ζ‐potential.^[^ ^96^, ^97^ ^]^ This implies that the ζ‐potential of the CuO NPs in solution is a functional surface property that is highly sensitive to the actual surface state of the dry CuO NPs. This is convincingly demonstrated by plotting the measured ζ‐potential of the as‐received, as‐washed, aged and surface‐treated CuO NPs in ethanol solution as a function of either the respective FWHM of the Cu 2*p*_3/2_ main peak or the resolved fraction of (sub)surface $O_{\mathrm{surf}}^{\delta -}$ species in **Figure** **11**a,b, respectively.

The measured ζ‐potential of the as‐received CuO NPs equals (14±3) mV, which lies well below the typical threshold for a stable CuO NP dispersion in ethanol solvent (as confirmed in this study), indicating aggregation and agglomeration due to high attractive forces. The ζ‐potential of the CuO NPs is significantly increased after the washing treatment, as well as after the ozonation and air‐annealing treatment, reaching values of (111±23), (100±21), and (124±26) mV, respectively. This suggests a strongly modified interaction between the CuO NPs surface and ethanol molecules for a more pristine CuO surface state. After 1 year of aging in air, the ζ‐potential of the as‐washed state decreases to (59±12) mV, confirming that aging impacts the surface properties of CuO NPs. In particular, the as‐washed and surface‐treated CuO NPs all have relatively low fractions of $O_{\mathrm{surf}}^{\delta -}$ species, which should result in a more defined space charge region at the NP surface. In other words, the introduction of defective O species in the NP subsurface region extends the width of the space charge region of the oxide CuO NPs, resulting in a lower ζ‐potential.^[^ ^96^, ^97^ ^]^ As discussed in Section 2.5, the as‐washed and ozonated CuO NPs still have a minor contribution of Cu(I) valence states at their surface, which apparently results in slightly higher ζ‐potential values as compared to an all‐CuO (II) surface state after air‐annealing.

EPD of micrometer‐thick nanoporous CuO coatings could only be successfully performed and reproduced using as‐washed and surface‐treated CuO NPs, as illustrated in Figure S10.1 (Supporting Information). Any aging of the treated CuO NPs in air resulted in an irreproducible EPD process. XPS analysis of the thus‐deposited CuO coatings (using an as‐washed NP state) was performed to detect possible chemical modifications of the NP surface state by reaction with ethanol: see Figure S10.2 (Supporting Information). The resulting Cu 2 p_3/2_ peak maintained its position at a BE of 933.3eV with the characteristic two‐peak maxima of the satellite structure being preserved. Hence, the surface state of CuO NPs is not noticeably affected by its dispersion in ethanol solvent and subsequent EPD.

The findings in this study underline the importance of establishing well‐defined surface treatments of oxide NPs to correct for inevitable aging effects and thereby extend the NP shelf‐life. Our findings and surface treatment strategies may thus contribute to a broader application of oxide NPs for innovative nanoparticle‐based technologies, like nanoporous oxide membranes, catalysts, functional coatings, and batteries.

## 3 Conclusion

In this study, we have systematically investigated the surface chemistry and dispersion characteristics of aged and surface‐treated CuO NPs. A robust qualitative XPS analysis approach was established to track cumulative changes in the surface chemistry of CuO NPs during long‐term air aging and after subsequent surface treatments. It was found that the Cu 2*p*_3/2_ main peak and its satellite serve as highly sensitive fingerprints for detecting subtle changes in the NP surface state. Atmospheric aging of the CuO NPs proceeds by hydroxylation with concurrent formation of organic‐O surface species, which induce a defective oxide subsurface region by adsorbate‐induced surface reconstructions. The aging process complies with a gradual transformation of CuO toward copper hydroxide and malachite‐like phases, as preferred by (bulk and interface) thermodynamics.

Sequential washing of aged CuO NPs with ethanol and MilliQ water effectively removes adsorbed impurities, thereby largely restoring the pristine surface state, although a small fraction of reduced Cu (I) valence states resides. Post‐washing treatments, including annealing in synthetic air and exposure to ozone, further contribute to obtain well‐defined CuO surface states. Notably, air‐annealing at 300°C not only further decreases the amount of adventitious carbon species, but also results in a complete annihilation (i.e., thermally‐activated oxidation) of Cu (I) valence states. In contrast, the room‐temperature ozonation treatment only acts on the outer surface and even further enhances the surface fraction of Cu (I) species with respect to the as‐washed state.

ζ‐potential measurements of aged and surface‐treated CuO NP dispersions in ethanol illustrate a delicate interplay between the surface state of the dry NPs and their colloidal stability. The ζ‐potential values correlate strongly with the structural and chemical heterogeneity of the NP surface. Aged particles have an increased concentration of Adv‐C surface species, as well as defective O subsurface species, resulting in a decreased ζ‐potential and colloidal destabilization. As‐washed and surface‐treated CuO NPs exhibit a much more uniform surface state, resulting in much higher ζ‐potential values.

We thus demonstrate how by tuning only the surface state of the CuO nanopowder, the ζ‐potential of the dispersed NPs can be adjusted for advanced manufacturing of nanoporous films by electrophoretic deposition. Up to date, usually surfactants have to be added to achieve similar reproducible results. We believe that this is a significant advancement in the field of nanopowder‐based manufacturing technologies for a broad range of applications, such as catalysis, sensing, batteries, energy conversion, joining.

## 4 Experimental Section

### Materials and Analysis

The current study employs two commercial CuO nanopowders, purchased from Thermo Scientific (catalogue number 044663.18) and Sigma Aldrich (SA; catalogue number 544868), referred to as **TS‐NP** and **SA‐NP**. Batches of SA‐NP were switched to a new one once the previous batch ran out. Small morphological differences between different batches are discussed in Section 2.1. Copper hydroxide (Cu(OH)_2_) reference powder was purchased from Alfa Aesar (tech. 94%). A bulk CuO thin‐film reference was prepared by thermal oxidation of a magnetron‐sputtered Cu metal layer at 350°C in synthetic air (20vol.% – O_2_/Ar) at normal pressure, as detailed in ref. [83]: see also Section S2 (Supporting Information). Furthermore, a malachite Cu_2_(OH)_2_CO_3_ reference powder was synthesized in‐house using the sol–gel method, following the procedure outlined in ref. [27]: see Section S3 (Supporting Information).

The bulk phase constitution of all samples was confirmed by XRD analysis using a Bruker D8 Discover with a Cu–Kα radiation source operating in a Bragg–Brentano geometry. The averaged crystallite size was estimated by Williamson–Hall analysis.^[^ ^98^ ^]^ The morphology of the initial nanopowders was investigated using a ZEISS GeminiSEM 460 scanning electron microscope (SEM) equipped with an InLens SE detector for high‐resolution imaging. Furthermore, the specific surface areas (SSAs) of the TS and SA CuO nanopowders were determined by the Brunauer–Emmett–Teller (BET) method using a 5‐point N_2_ adsorption BET isotherm, as measured with a Belsorp Mini X from Microtrac. Prior to the BET analysis, the powders were degassed for 2h at 180°C and 10Pa. The ζ‐potentials of dispersed CuO NPs in ethanol solvent for concentrations in the range of 0.5‐0.7w.% were measured by the multi‐frequency (300kHz–3MHz) electroacoustic method using a Zeta Probe instrument (Colloidal Dynamics Inc., North Attleboro). The Zeta Probe employs a theoretical model developed by O'Brien et al.^[^ ^99^ ^]^ to relate the measured dynamic mobility to the ζ‐potential in concentrated colloids.

The chemical state of the CuO NP surfaces after aging, as well as after different surface treatments (see Section Washing, Aging and Surface Treatments), was studied in great detail by XPS analysis using a PHI Quantes spectrometer (ULVAC‐PHI) with a monochromatic Al‐Kα X‐ray source (*h*ν = 1486.6 eV, power 51W, beam diameter 200 µm, detection angle of 45°). The energy scale of the hemispherical analyzer was calibrated according to ISO standard 15472 (2nd edition, 2010‐05‐01) by referencing the Au 4*f*_7/2_, Ag 3*d*_5/2_ and Cu 2*p*_3/2_ main peaks to the recommended binding energy (BE) positions of 83.96, 368.21, and 932.62 eV, respectively (as in‐situ measured for the sputter‐cleaned, high‐purity metal references). The nanopowders were pressed on a sticky carbon tape (as attached to a stainless‐steel holder) in an under‐pressurized Ar glovebox system (O_2_⩽0.1ppm, H_2_O⩽0.2ppm) and transferred without intermediate air exposure for subsequent XPS analysis. The UHV chamber of the XPS instrument is directly coupled to the glove box to safely accept the dry nanopowder samples through a load‐lock system. The sticky carbon tape gives a characteristic signal from Si in the XPS analysis, which is not detected if the selected XPS analysis area is densely covered with the manually gently pressed nanopowder; this criterion was used for selecting the XPS analysis areas (having a diameter of 200 µm, see above). Survey scans of the selected areas were acquired with a pass energy of 224eV and a step size of 0.8eV. Next, high‐resolution scans of the Cu 2*p*, C 1*s*, and O 1*s* regions were measured with a pass energy of 69eV and a step size of 0.125eV. Charge neutralization during XPS analysis was accomplished by dual‐beam charge neutralization, employing low‐energy electron and Ar ion beams (1‐V bias, 20‐μA current).

### Washing, Aging and Surface Treatments

The as‐received CuO nanopowders were stored in their original vessel in an Ar glove box (O_2_⩽0.6ppm, H_2_O⩽0.2ppm) directly upon delivery, thus preventing uncontrolled surface reactions with the laboratory environment during in‐house storage (denoted as **“as‐received”**). Notably, the as‐received nanopowder stock might have already aged to some extent during synthesis, storage, and shipment. For all studies performed in this work, individual batches were sampled from the as‐received nanopowder stock under Ar. Next, as a first step, possible contaminants introduced from the synthesis route (presumably wet‐chemical synthesis;^[^ ^27^ ^]^ see Section 2.1) were removed by successively washing the sampled nanopowder batch with MilliQ water (with a conductivity of σ = 0.055μSm cm^−1^ at 25°C) and absolute ethanol (Sigma Aldrich, ⩾99.8%). For each washing treatment, ≈5mL of CuO nanopowder was weighted in a 50 mL conical centrifuge tube and filled to 35mL with MilliQ water or ethanol. The tube was placed in an ultrasonic bath for 10min (with frequency and power of 37 kHz and 100%, respectively), after which the sample was sedimented using a centrifuge (7s ramp up to 10 000 rpm (RCF of 13751× g), hold for 10min followed by 7s ramp down). The sonication process was carried out in an ice bath to avoid possible reduction of CuO by ethanol (see Section 2.4). The above washing procedure was repeated six times (i.e., ending with an absolute ethanol washing step), after which the washed nanopowder was dried by evacuation in a desiccator overnight.

The **“annealed”** surface treatment consisted of a subsequent annealing step of the as‐washed nanopowder in a tube furnace under synthetic air flow (at a flow rate of 0.3 L m^−1^ in 20vol.% – O_2_/N_2_) at 300°C. Notably, a short 25–35°C overshoot above the target temperature occurred; see Figure S4.1 (Supporting Information). The annealing profile was performed with a ramp‐up rate of 10°C min^−1^, a dwell time of 10min at the target annealing temperature, followed by a slow self‐cooling stage aided by a continuing gas flow.

The **“ozonated”** surface treatment involved a room‐temperature treatment of the as‐washed nanopowder in an ozonated ambient gas atmosphere, as generated by UV illumination of air using a Digital UV Ozone System from Novascan Technologies (PSD Pro Series, USA). The washed CuO nanopowder was held in an alumina boat and placed in a chamber at a distance of 35mm from the UV lamp. The humidity during the ozone treatment was artificially increased by placing a beaker with 15 mL of MilliQ water in the chamber. The ozonated surface treatment was performed for 30 min, while briefly opening the chamber every 10 min to gently shake the boat and thereby mix the nanopowder to achieve a more uniform exposure of the NPs to ozone.

The **“aged”** nanopowders should reflect the uncontrolled change of the surface state of the as‐washed CuO nanopowder when stored under ambient (i.e., in humid air) conditions over time. To ensure roughly constant ambient aging conditions, the as‐washed CuO nanopowders were stored in a closed centrifuge tube within the fume hood, with the tube being periodically opened for the purpose of analysis. This approach ensures a consistent baseline for evaluating the effects of aging under laboratory conditions. Any deviation from the initial as‐washed surface state will be referred to as aging.

### XPS Data Evaluation

All measured XPS spectra were analyzed using the Casa XPS software (Version 2.3.25PR1.0). As a first step, the high‐resolution Cu 2*p*_3/2_ − 2*p*_1/2_, C 1*s*, and O 1*s* spectra for each XPS measurement were charge‐corrected by peak fitting the respective C 1*s* spectrum and subsequently adjusting the corresponding binding energy (BE) scale such that the lowest BE component of the C 1s spectrum (as attributed to alkyl‐type C─C/C─H surface species: see Section 2.4) matches the recommended standardized value of 284.8 eV.^[^ ^60^ ^]^ Spectral reconstruction of the C 1*s* and O 1*s* regions was performed by linear‐least‐squares fitting of the Shirley‐background corrected spectra with one or more synthetic Gaussian–Lorentzian (GL) peak components. For the C 1*s* spectral reconstruction, a line shape of GL(30) was used while fitting a single value of the Full Width at Half Maximum (FWHM) for all main peaks. For the O 1*s* spectral reconstruction, a line shape of GL(70) was used while fitting a single FWHM for all surface species (see Section 2.4) and a separate FWHM for O in the bulk (CuO) lattice. See Table 1 for more details. A Python script was developed for automatically evaluating the relative BE positions and FWHMs of the Cu 2*p*_3/2_ main peak and its respective satellite from the Shirley‐background corrected spectra, as exemplified in Section S6 (Supporting Information).

## Conflict of Interest

The authors declare no conflict of interest.

## Author Contributions

A.S.B. was responsible for investigation, methodology, formal analysis, visualization, data curation, and writing of the original draft. B.R. contributed to validation, supervision, and writing‐review and editing. C.C. performed investigation related to thin film references, XPS and XRD validation, and writing‐review and editing. L.P.H.J. contributed to conceptualization, funding acquisition, project administration, resources, validation, supervision, and writing–review and editing.

## Supporting information

Supporting Information

## Data Availability

The data that support the findings of this study are available from the corresponding author upon reasonable request.
