# Supplementary material for: Tuning the Surface State and Zeta Potential of Aged CuO Nanoparticles by Additive‐Free Surface Treatments as Guided by XPS
Source: Adv Mater. 2025 Oct 26;38(5):e13030. doi: 10.1002/adma.202513030 (PMC12822534; doi:10.1002/adma.202513030)
Supplement: Supplementary file 1 — Supporting Information [file ADMA-38-e13030-s001.pdf]

ADVANCED MATERIALS

Supporting Information

for *Adv. Mater.*, DOI 10.1002/adma.202513030

Tuning the Surface State and Zeta Potential of Aged CuO Nanoparticles by Additive-Free Surface Treatments as Guided by XPS

*Anastasia S. Batenkova, Bastian Rheingans, Claudia Cancellieri and Lars P.H. Jeurgens**

SUPPLEMENTARY MATERIAL
Tuning the Surface State and Zeta Potential of aged CuO Nanoparticles by Additive-Free Surface Treatments as Guided by XPS

Anastasia Batenkova[a,b], Bastian Rheingans[a], Claudia Cancellieri[a], Lars P.H. Jeurgens[a,*]

[a] *Empa - Swiss Federal Laboratories for Materials Science and Technology, Laboratory for Joining Technologies and Corrosion, Dübendorf, Switzerland*
[b] *ETHZ - Federal Institute of Technology Zurich, Department of Chemistry and Applied Biosciences, Zurich, Switzerland*

S1. TEM images of CuO NP

The nanopowders were dispersed in ethanol and then ultrasonically treated (as for the washing procedure). Next the NP dispersion was drop-casted on a transparent TEM grid before performing TEM analysis using a JEOL JEM2200fs operated at 200 kV.

* Corresponding author: lars.jeurgens@empa.ch

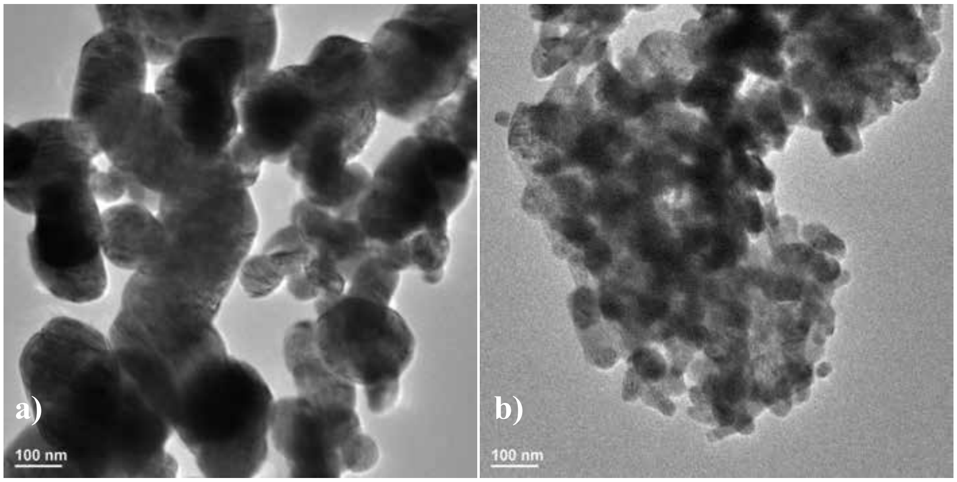

Figure S1.1: TEM micrographs of the as-received CuO nanopowders: (a) Thermo Scientific (TS) and (b) Sigma Aldrich (SA). The TEM analysis evidences agglomeration/aggregation, as well as the presence of individual nanoparticles with sizes exceeding 100 nm.

S2. CuO Thin-Film Reference

A CuO film reference was prepared by thermal oxidation of the 300 nm-thick Cu films in synthetic air (20 vol.% – O_2/Ar) at 350 °C. A detailed description of the CuO oxidation procedure is given in Ref. [2]. The single-phase constitution (i.e. CuO) was confirmed by XRD analysis, as shown in Fig. **S2**.1. Except for adventitious carbon, no other surface contaminations were detected by XPS: see Fig. **S2**.2.

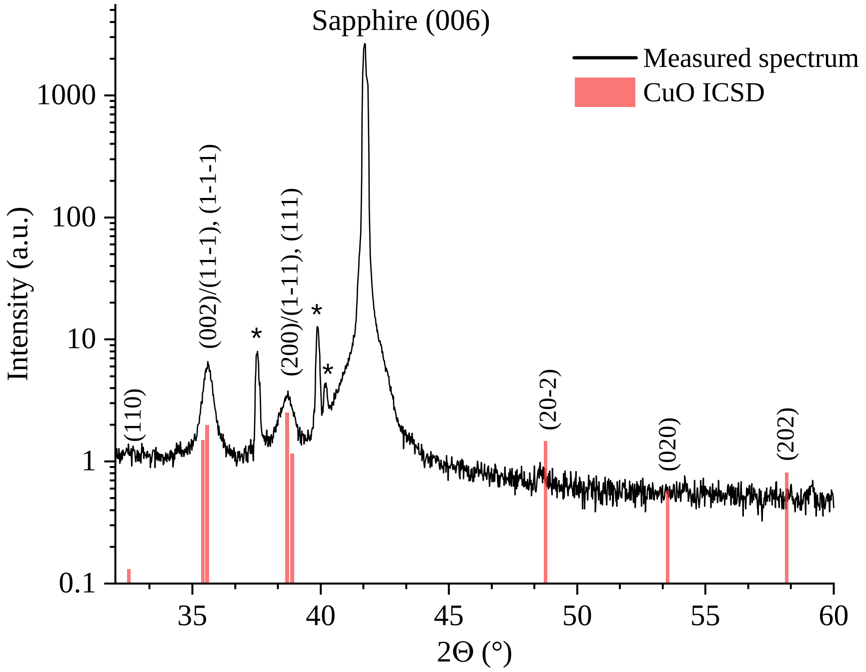

Figure S2.1: XRD diffractogram of the CuO thin-film reference. Reflections arising from non-monochromatic X-ray radiation are marked with asterisks "*". The default reflections for CuO with respective Miller indices from the ICSD database are indicated in red

.

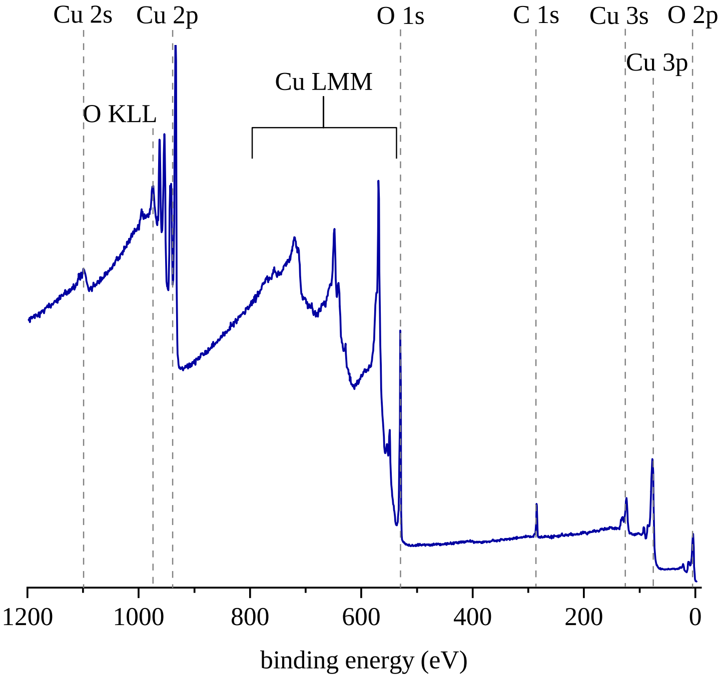

Figure S2.2: Measured XPS survey spectrum of the CuO thin-film reference.

S3. Malachite reference

A malachite, $Cu_2(OH)_2CO_3$, reference was prepared in-house according to the procedure described in Ref. [1]. Except for adventitious carbon, no other surface contaminants were detected by XPS: see Fig. **S3**.1. Constrained curve-fitting of the measured C 1 *s* and O 1 *s* spectra was performed to resolve the peak positions and chemical shifts of the C−C / C−H, organic-O, −OH, $-CO_3$ components: see Figs. **S3**.2(a,b) and Table 1 in Section 3.3 of the paper. The measured Cu 2 *p* region is shown in Figs. **S3**.2(c), as reported in Table 2 in Section 3.3.

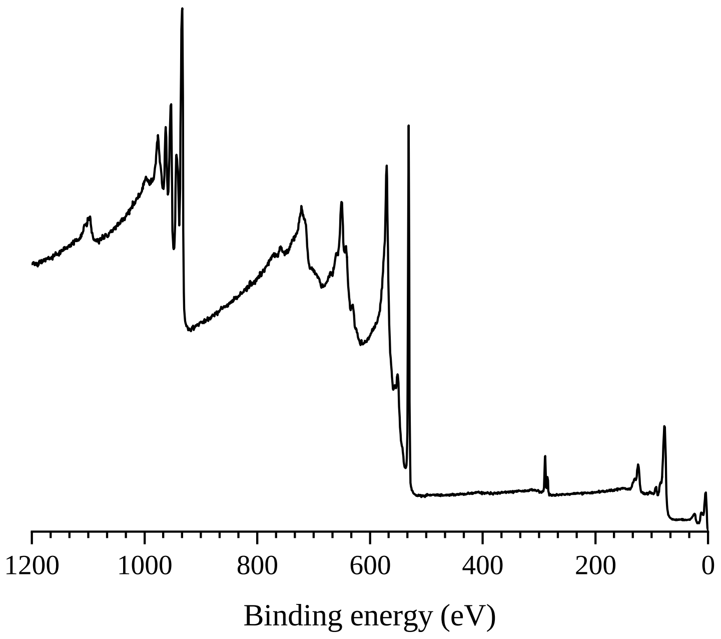

Figure S3.1: XPS survey spectrum of the as-prepared malachite reference.

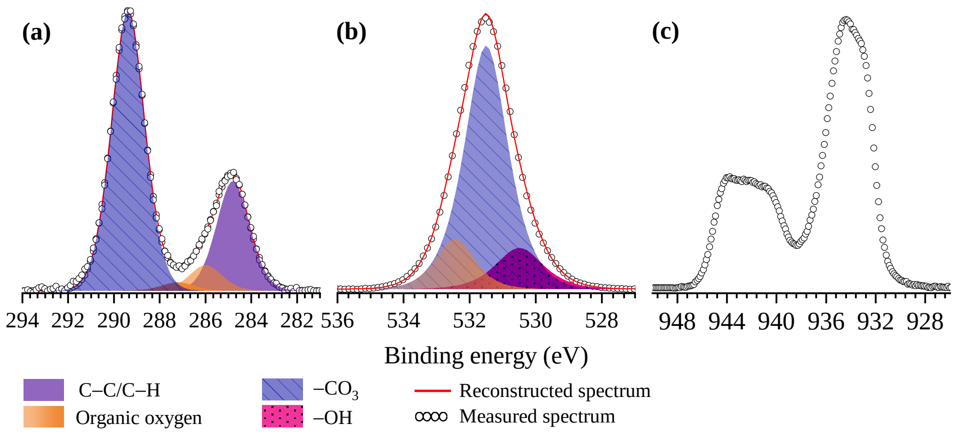

Figure S3.2: Spectral reconstruction of (**a**) the C 1 s region and the (**b**) O 1 s region, as measured from the malachite reference by XPS. (**c**) Measured Cu 2 p region corresponding to (a,b).

S4. Temperature profile of the thermal annealing treatment

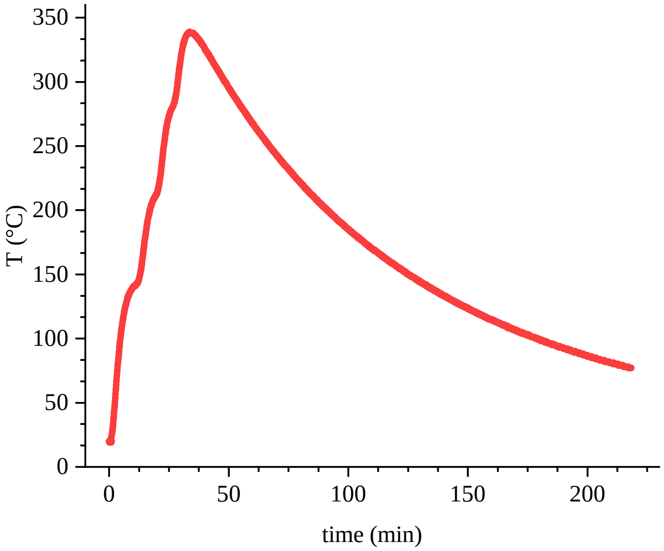

Figure S4.1: Heating curve pertaining to the thermal annealing of the CuO NP at 300°C in synthetic air.

S5. Investigation of possible Differential Charging Issues

To experimentally investigate the effectiveness of the dual-beam floodgun in suppressing differential charging during XPS analysis, an identical XPS measurement was performed on different positions of a given CuO nanopowder sample, while either grounding or floating the sample with respect to the stage. Samples were mounted using three different methods: non-conductive 3M tape, non-conductive 3M tape with a conducting mask (with a center hole about 3 times larger than the analysis area), and conductive carbon tape. Next, the same measurement was repeated at different locations on the same sample. Exemplary spectral reconstructions for thus-measured O 1 s spectra are shown in Fig. **S5**.1. It follows that the chemical shifts between the resolved peak components are constant within the experimental error (of ±0.1 eV) for the different sample mounting methods and selected analysis positions. The same holds for the FWHMs of the resolved peak components. Common signs of differential charging, such as (asymmetric) skewing of the resolved peak components and ”unidentified” ghost peaks, were not observed.

However, depending on the actual NP packing density in the probed analysis volume, the measured ratio of the photoelectron intensity originating from surface and core regions of the NP assembly was found to vary: e.g. slight variations of the bulk spectral contribution, O_{bulk}, with respect to all other surface contributions in Fig. **S5**.1. This is caused by the fact that more surface to bulk is probed for less densely packed NP regions (see Sec. 3.2). Photoelectrons emitted from core and surface regions of the CuO NP assembly will experience difference inelastic scattering cross-sections to reach the analyzer, depending on the size distribution and packing density of the NPs, as well as on varying degrees of surface contamination by adventitious carbon species throughout the porous structure. Hence, for a given nanopowder sample, the absolute photoelectron intensity, as well as the probed average surface-to-volume ratio of the NP assembly will depend on the selected position and area for the XPS analysis (see Fig. 3). To approximately correct for variations in absolute photoelectron intensity, depending on the selected position for XPS analysis, all recorded spectra were normalized by the integrated intensity below the Shirley-background-corrected peak envelop: see e.g. Fig. **S5**.1. Still the relative signal intensities from surface and core regions of the NP assembly will vary depending on the area and position of the XPS measurement, which hinders a reliable quantification of the (absolute) composition of e.g. surface and bulk species on the basis of the reconstructed Cu 2p, C 1s and O 1s spectral contributions (Fig. 4).

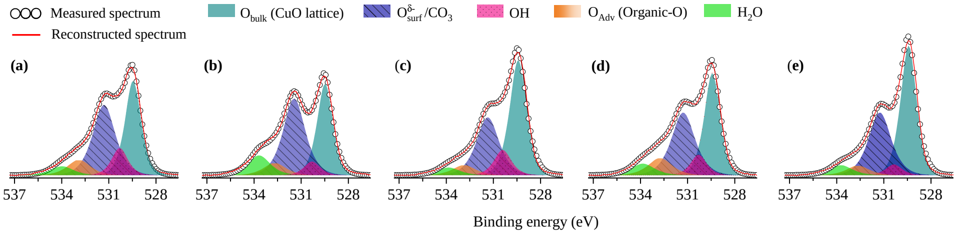

Figure S5.1: Reconstructed O 1s regions as measured from the CuO SA-NP after washing and O_3 exposure. (**a**) as mounted on the non-conductive 3M tape with a conducting mask; (**b**), (**c**) as mounted on a non-conductive 3M tape for two selected positions on the same sample; (**d**), (**e**) as mounted on a conductive carbon tape for two selected positions on the same sample.

S6. Evaluation procedure for Cu $2p_{3/2}$ region

A Python code was developed to automatically evaluate characteristic features of the Cu $2p_{3/2}$ main peak and its satellite. In a first step, the BE

positions of the local minima (see green dots in Fig. **S6**.1) were identified to define the BE ranges between the main peak and the onset and end of the satellite peak. The range of the main peak is determined as the energy range between `min_main` and `local_min`. The range of the satellite peak is defined as the energy range between `local_min` and `max_satellite`. Next, a Shirley background is subtracted over the energy range from `min_main` to `max_satellite`. Next, to determine the maximum intensity of the satellite peak, the script calculates the average intensity values between `top_min` and `top_max`. If the satellite peak does not exhibit a well-defined two-peak fine structure, the program takes the average of the maximum intensities in the predefined BE ranges from 939 – 942 eV for `top_min` and from 943 – 945 eV for `top_max` range. The spectrum is evaluated iteratively for all identified peaks. For each peak, an interpolation algorithm refines the data grid to improve precision. Finally, the code analyzes the low and high BE sides of the main and satellite peaks to identify BE values corresponding to one half of the maximum intensity of the determined peak maxima at opposite flanks of the peak. The Full Width at Half Maximum (FWHM) for each peak corresponds to the energy separation of the thus-determined BE values at opposite flanks of the respective peak.

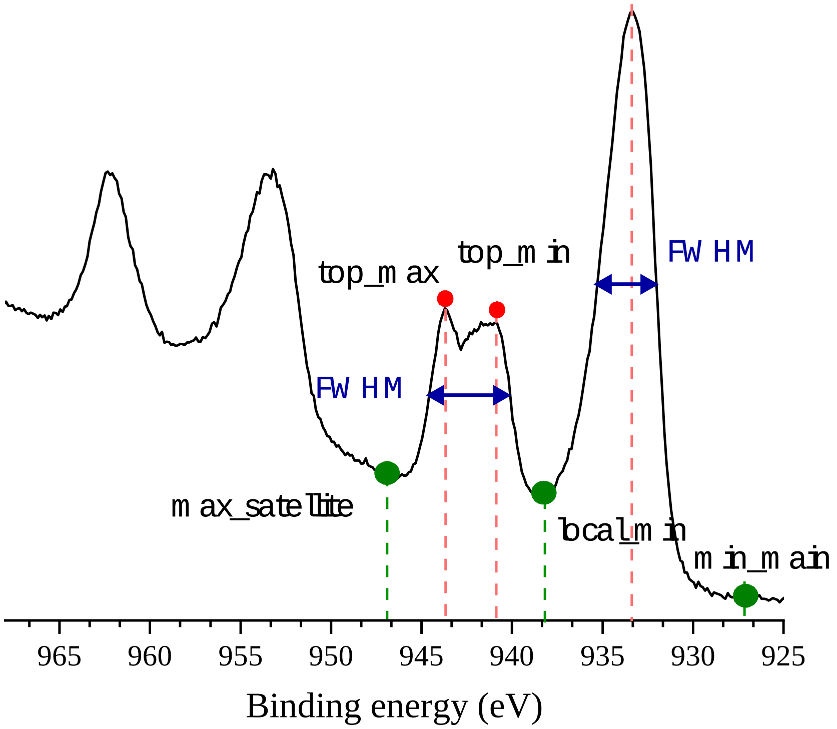

Figure S6.1: The sketch shows an example of the fitting procedure of the Cu $2p_{3/2}$ main peak and its satellite. Green circles and dashed lines represent the calculated intensity minima and BE values, respectively. Red circles and dashed lines show the determined peak maxima of the main and satellite peaks. For the main line, the actual intensity maximum is used to calculate the FWHM, while for the satellite, the average intensity of the doublet is used. See text for details.

S7. Measured Cu LMM spectra

Measured XPS spectra of the Cu LMM Auger line for different surface states of the CuO NP, as well as for a Cu metal, Cu_2O and CuO thin films, are shown in Fig. **S7**.1. The reference spectrum of metallic Cu is characterized by a distinct peak at 921.3 eV (kinetic energy, KE), which is absent in all CuO samples studied. We can thus exclude the possibility of Cu (II) being fully reduced to metallic Cu (0) during washing, aging, or surface treatments. A complete reduction of Cu (II) to Cu (I) can also be ruled out, as this would lead to a pronounced shift of the main peak towards lower KEs (see dashed pink line for Cu_2O) and the appearance of a shoulder at the higher KE side of the main peak (at around 922 eV) [75]. The as-received

and treated nanopowders do not exhibit such a shift of the Cu LMM Auger line nor a distinct shoulder at around 922 eV. Interestingly, a noticeable shift of the Cu LMM Auger line to lower KE towards the peak position for Malachite is observed after 2-years aging (orange dash-dot line), which hints at a sluggish solid-state transformation of the nanopowder into Malachite during prolonged aging. We thus also rule out a significant reduction of Cu (II) to Cu (I) during aging/washing/treatment. Instead, all studied CuO nanopowders show peak positions at higher kinetic energies, consistent with a dominance of Cu (II) valence states in the as-received, aged and treated states.

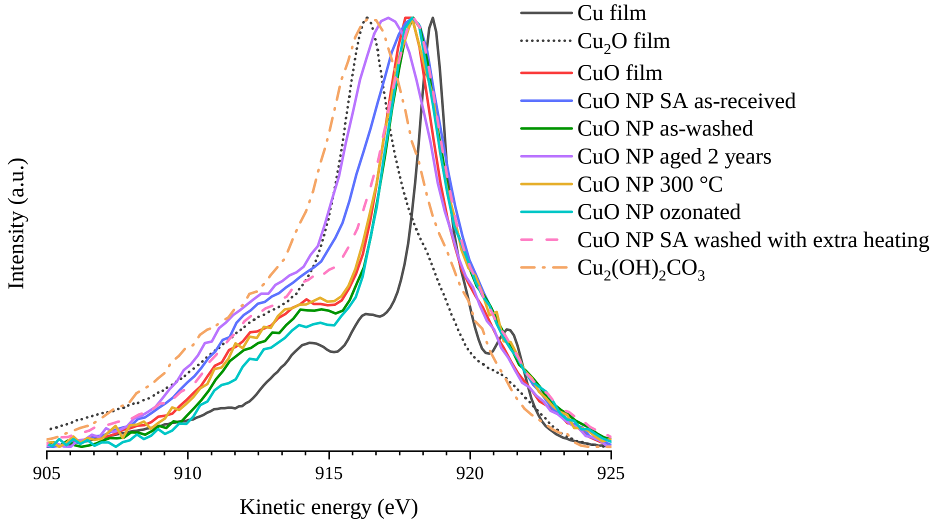

Figure S7.1: Measured XPS spectra of the Cu LMM Auger line for different surface states of the CuO NP, as well as for a Cu metal, Cu_2O

and CuO thin films. The spectra have been normalized and Shirley-background corrected to ease visual comparison.

S8. XRD of the CuO NPs before and after surface treatment

XRD analysis of the studied nanopowders confirms a bulk cupric oxide phase for all NP surface states studied: see Fig. **S8**.1. Additional XRD measurements were carried out to exclude any phase transformations induced by washing. Since washing with ethanol can cause a partial reduction of Cu (II), the washing was carried out under extreme conditions: The US bath was heated to 45 °C, which facilitated the red/ox reaction. However,

still the XRD analysis revealed only a bulk cupric oxide phase (no indication of e.g. Cu_2O): see Fig. **S8**.1.

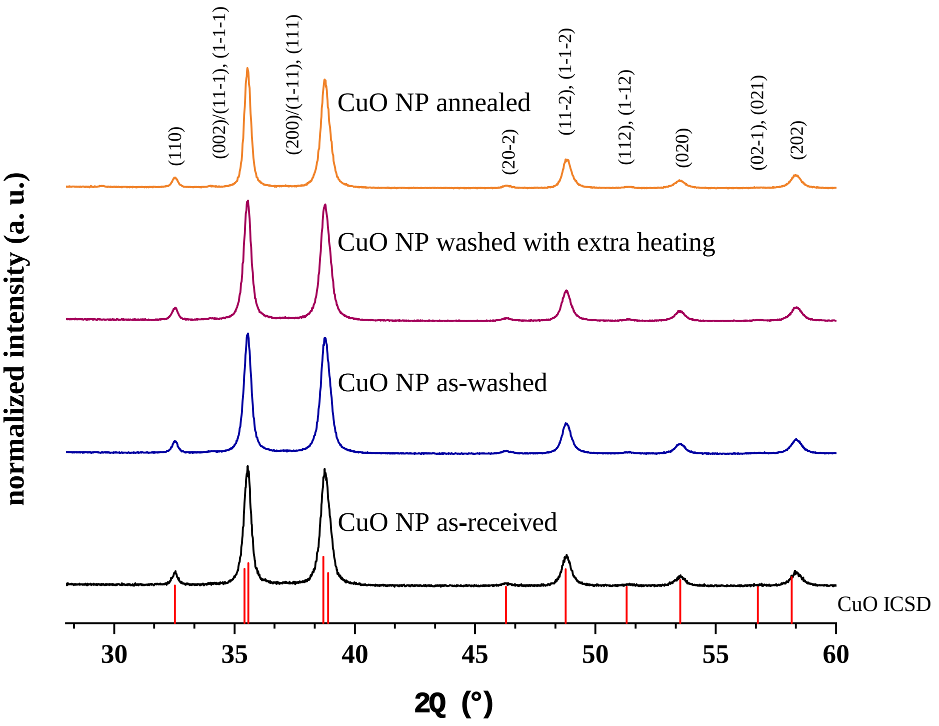

Figure S8.1: XRD diffractograms of the as-received SA-NP, as-washed and surface-treated CuO NPs. The default reflections for CuO according to the ICSD database are indicated by the red bars and respective Miller indices

.

The average primary crystallite size of the as-received, treated, and aged nanopowders was estimated using the Williamson-Hall (W-H) analysis; see Table 1. The treated nanopowders have a smaller average crystallite size than the as-received ones, presumably due to ultrasonication during washing and the removal of larger particles/aggregates when changing vials.

**SA-Nanopowder**	**Average crystallite size** [nm]
CuO-TS as-received	60
CuO-SA as-received	50
CuO as-washed	33
CuO aged 1 year	35
CuO annealed	36
CuO ozonated	39

Table 1: The average crystallite size of the as-received, treated, and aged CuO NPs as estimated using the W-H analysis.

S9. Measured Cu 2 p region of the studied nanopowders by XPS

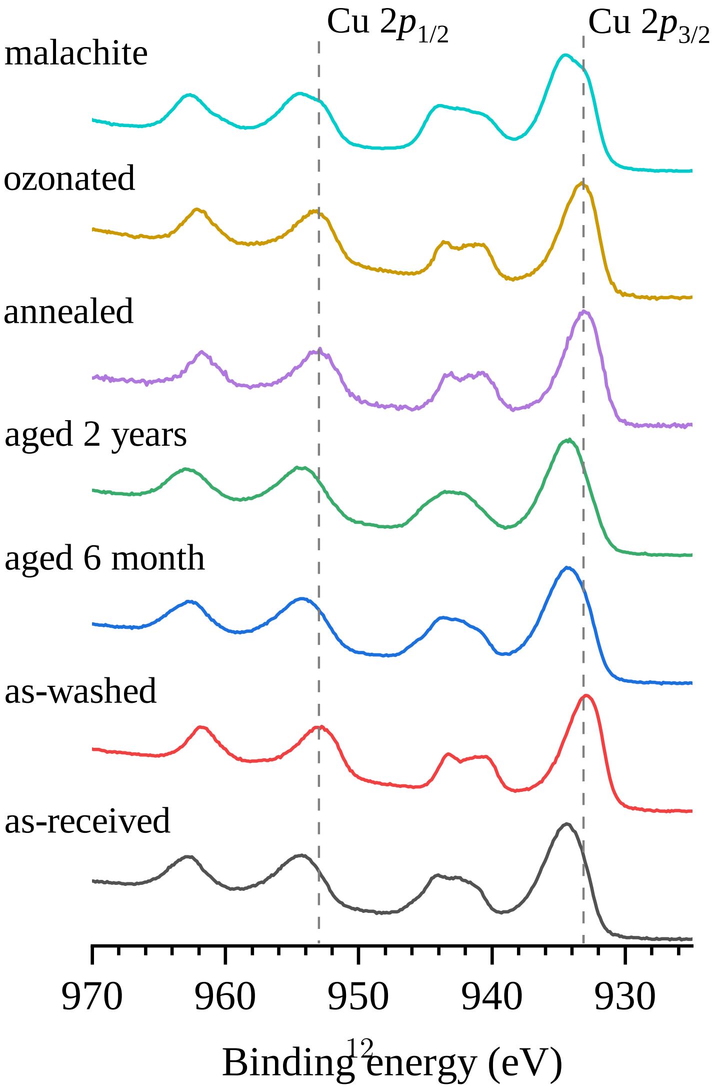

Figure S9.1: The Cu 2 p region, showing the Cu $2p_{3/2} - 2p_{1/2}$ doublet, as recorded from the nanopowders by XPS. The splitting energy of the Cu $2p_{3/2} - 2p_{1/2}$ doublet is about (20.0 ± 0.2) eV for all nanopowders studied. All spectra were normalized with respect to the maximum peak intensity

S10. Electrophoretic deposition (EPD) of CuO nanoparticles

EPD was successfully performed at 25 V from a dispersion with as-washed CuO NP (concentration of $5\,g\,L^{-1}$) in ethanol solvent, following the procedure as detailed in Ref. [1]. A cross-sectional SEM micrograph of the nanoporous coating is shown in Fig. **S10**.1.

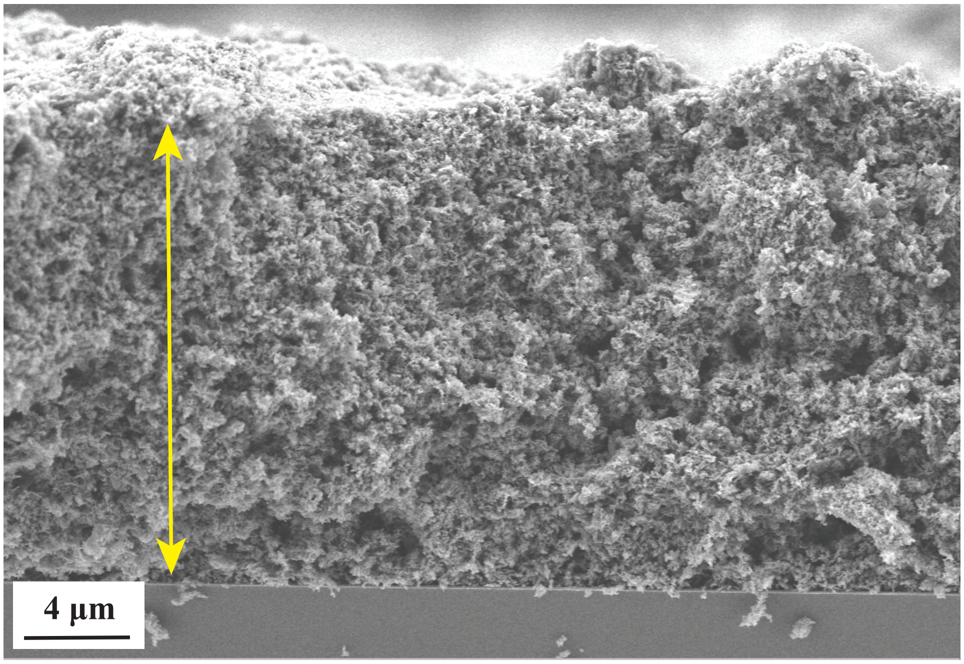

Figure S10.1: Cross-sectional SEM micrograph of a nanoporous CuO coating as obtained by electrophoretically deposition. The coating thickness is about ≈20 μm

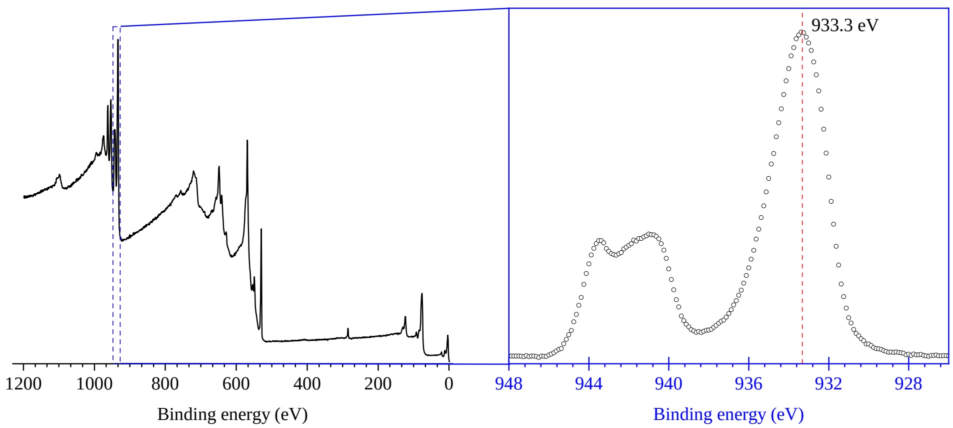

Figure S10.2: Measured XPS survey and Cu 2 p spectra of the CuO coating, as fabricated by electrophoretic deposition from a CuO NP dispersion in ethanol solvent. The Cu 2 $p_{3/2}$ peak maintained its position at a BE of 933.3 eV with the characteristic two-peak maxima of the satellite structure being preserved. Hence, the surface state of CuO NPs is not noticeably affected by its dispersion in ethanol solvent and subsequent EPD.

References

[1] Lars Dörner et al. "Cost-effective sol-gel synthesis of porous CuO nanoparticle aggregates with tunable specific surface area". In: *Sci. Rep.* 9.1 (Aug. 2019). ISSN: 2045-2322. DOI: 10.1038/s41598-019-48020-8.

[2] Yeliz Unutulmazsoy et al. "Reduction of thermally grown single-phase CuO and Cu2O thin films by in-situ time-resolved XRD". In: *Appl. Surf. Sci.* 588 (June 2022), p. 152896. ISSN: 0169-4332. DOI: 10.1016/j.apsusc.2022.152896.
